# Genomic Evidence of Rapid and Stable Adaptive Oscillations over Seasonal Time Scales in Drosophila

**DOI:** 10.1371/journal.pgen.1004775

**Published:** 2014-11-06

**Authors:** Alan O. Bergland, Emily L. Behrman, Katherine R. O'Brien, Paul S. Schmidt, Dmitri A. Petrov

**Affiliations:** 1 Department of Biology, Stanford University, Stanford, California, United States of America; 2 Department of Biology, University of Pennsylvania, Philadelphia, Pennsylvania, United States of America; University of Texas at Austin, United States of America

## Abstract

In many species, genomic data have revealed pervasive adaptive evolution indicated by the fixation of beneficial alleles. However, when selection pressures are highly variable along a species' range or through time adaptive alleles may persist at intermediate frequencies for long periods. So called “balanced polymorphisms” have long been understood to be an important component of standing genetic variation, yet direct evidence of the strength of balancing selection and the stability and prevalence of balanced polymorphisms has remained elusive. We hypothesized that environmental fluctuations among seasons in a North American orchard would impose temporally variable selection on *Drosophila melanogaster* that would drive repeatable adaptive oscillations at balanced polymorphisms. We identified hundreds of polymorphisms whose frequency oscillates among seasons and argue that these loci are subject to strong, temporally variable selection. We show that these polymorphisms respond to acute and persistent changes in climate and are associated in predictable ways with seasonally variable phenotypes. In addition, our results suggest that adaptively oscillating polymorphisms are likely millions of years old, with some possibly predating the divergence between *D. melanogaster* and *D. simulans*. Taken together, our results are consistent with a model of balancing selection wherein rapid temporal fluctuations in climate over generational time promotes adaptive genetic diversity at loci underlying polygenic variation in fitness related phenotypes.

## Author Summary

Herein, we investigate the genomic basis of rapid adaptive evolution in response to seasonal fluctuations in the environment. We identify hundreds of polymorphisms (seasonal SNPs) that undergo dramatic shifts in allele frequency – on average between 40 and 60% – and oscillate between seasons repeatedly over multiple years, likely inducing high levels of genome-wide genetic differentiation. We provide evidence that seasonal SNPs are functional, being both sensitive to an acute frost event and associated with two stress tolerance traits. Finally, we show that some seasonal SNPs are possibly ancient balanced polymorphisms. Taken together, our results suggest that environmental heterogeneity can promote the long-term persistence of functional polymorphisms within populations that fuels fast directional adaptive response at any one time.

## Introduction

All organisms live in environments that vary through time and such environmental heterogeneity can impose highly variable selection pressures on populations. In this situation, an allele may be beneficial during one environmental regime and subsequently deleterious during another. Such an allele would be subject to short bursts of directional selection, alternately being favored and disfavored. When this situation occurs in diploids, the heterozygote can have a higher geometric mean fitness than either homozygote and allelic variation at this locus could be maintained for long periods despite being subject to directional selection at any given time [1]–[8]. This situation is referred to as marginal overdominance and is a form of balancing selection.

There is substantial evidence for the maintenance of phenotypic and genetic variation by temporally variable selection in a variety of organisms. For instance, evolutionary response to rapid changes in selection pressures has been demonstrated for morphological and life-history traits in mammals [9], [10], birds [11]–[13], plants [14], invertebrates [15]–[24], and others (reviewed in [25], [26]). Chromosomal inversions and allozyme alleles in a variety of drosophilids vary among seasons [27]–[33] suggesting that these polymorphisms confer differential fitness in alternating seasons. Further, in some species of drosophilids, life-history [34], [35], morphological [36], [37] and stress tolerance traits [38], [39] also fluctuate seasonally suggesting that these traits respond to seasonal shifts in selection pressures.

Although theoretical models suggest that temporal variation in selection pressures can maintain fitness-related genetic variation in populations [1]–[8] and empirical evidence from a variety of species [9]–[39] demonstrates that variation in selection pressures over short time periods does alter phenotypes and allele frequencies, we still lack a basic understanding of many fundamental questions about the genetics and evolutionary history of alleles that undergo rapid adaptation in response to temporal variation in selection pressures. Specifically, we do not know how many loci respond to temporally variable selection within a population, the strength of selection at each locus, nor the effects of such strong selection on neutral genetic differentiation through time. We do not know whether adaptation at loci that respond to temporally variable selection is predictable nor do we know the relationship between loci that respond to temporally variable selection and spatially varying selection. Finally, it is unclear whether rapid adaptation to temporally variable selection pressures is primarily fueled by young alleles that constantly enter the population but cannot be maintained for long periods of time or, rather, by old alleles that have possibly been maintained by variable selection associated with environmental heterogeneity despite short bursts of strong directional selection.

To address these questions, we estimated allele frequencies genome-wide from samples of *D. melanogaster* individuals collected along a broad latitudinal cline in North America and in the spring and fall over three consecutive years in a single temperate orchard. We demonstrate that samples of flies collected in a single Pennsylvania orchard over the course of several years are as differentiated as populations separated by 5–10° latitude. We identify hundreds of polymorphisms that are subject to strong, temporally varying selection and argue that genetic draft [40] in the wake of rapid, multilocus adaptation is sufficient to explain the high degree of genetic turnover that we observe in this population over several years. We examine the genome-wide relationship between spatial and temporal variation in allele frequencies and find that spatial genetic differentiation, but not clinality *per se*, in allele frequency is a good predictor of temporal variation in allele frequency. Moreover, at SNPs subject to seasonal fluctuations in selection pressures, northern populations are more similar to spring populations than southern ones are. Next, we show that allele frequencies at SNPs subject to seasonal fluctuations in selection pressures become more ‘spring-like’ (i.e., they move towards the average spring frequency) immediately following a hard frost event and that seasonally variably SNPs tend to be associated with two seasonally variable phenotypes, chill coma recovery time and starvation tolerance. Finally, we demonstrate that some of the loci that respond to temporal variation in selection pressures are likely ancient, balanced polymorphisms that predate the split of *D. melanogaster* from its sister species, *D. simulans*. Taken together, our results are consistent with a model in which temporally variable selection maintains fitness-related genetic variation at hundreds of loci throughout the genome for millions of generations if not millions of years.

## Results/Discussion

### Genomic differentiation through time and space

**Figure 1:**
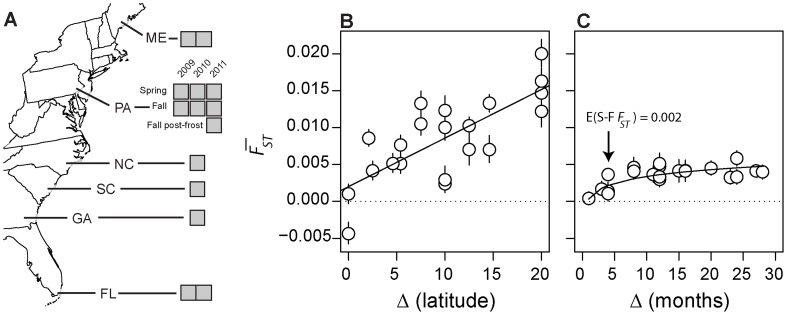
Experimental design and genomic turnover through time and space. (A) Map of sampling locations in North America used in this study. Grey boxes represent individual samples from each locale. Genome-wide differentiation among spatially (B) and temporally (C) separated samples, measured as genome-wide average *F_ST_* (y-axis). Lines represent the predicted value of *F_ST_* based on the linear (A; y = a+bx) and non-linear (B; y = ab^X^) regression. Note: Pennsylvanian samples are not represented in (B) and the negative *F_ST_* in (B) results from the conservative correction of heterozygosity [102], [103]. In addition, please note that there are four estimates of pairwise *F_ST_* between the two replicate Maine and Florida samples (corresponding to a difference in latitude of 20°) and that there are two estimates of *F_ST_* between each of the remaining clinal populations and each Maine and Florida replicate sample. Error bars represent 95% confidence intervals based on 500 blocked bootstrap samples of ∼2000 SNPs.

To test for the genomic signatures of balancing selection caused by seasonal fluctuations in selection pressures, we performed whole genome, pooled resequencing of samples of male flies collected in the spring and fall over three consecutive years (2009–2011) in a temperate, Pennsylvanian orchard. We contrast changes in allele frequencies through time with estimates of allele frequencies we made from five additional populations spanning Florida to Maine along the east coast of North America over a number of years (2003–2010) largely during periods of peak abundance of *D. melanogaster* (Fig. 1A, Table S1). From each population and time point, we sampled approximately 50–100 flies and resequenced each sample to average read depth of 20–200× coverage (Table S1, and see Text S1). Estimates of allele frequency using this sampling design have been shown to be highly accurate [40].

As a point of departure and to provide context for understanding the magnitude of genetic variation through the seasons, we first examined genetic differentiation along the cline (Fig. 1B, Fig. S1A). We calculated genome-wide average *F_ST_* among pairs of populations (excluding Pennsylvanian populations; hereafter ‘spatial *F_ST_*’) as well as the proportion of SNPs where average spatial *F_ST_* between a pair of populations is greater than expected by chance conditional on our sampling design and assuming panmixia using allele frequency estimates of 500,000 common polymorphisms (Table S1). Genome-wide average spatial *F_ST_* (Fig. 1B) as well as the proportion of SNPs where spatial *F_ST_* is greater than expected by chance (Fig. S1A) is positively correlated with geographic distance (*r* = 0.75; *p* = 7e-5), a pattern consistent with isolation by distance [41]. Pooled resequencing did identify polymorphisms in or near genes previously shown to be clinal in North American populations (see Text S1) demonstrating that clines are stable over multiple years. This suggests that populations sampled along the cline represent resident populations, and further confirms that our pooled resequencing design gives accurate estimates of allele frequencies [42].

Next, we calculated genome-wide average *F_ST_* between samples collected through time in the Pennsylvanian population (‘temporal *F_ST_*’) as well as the proportion of SNPs where average temporal *F_ST_* is greater than expected by chance given our sampling design and assuming no allele frequency change through time (Fig. 1C, Fig. S1B). Genome-wide average temporal *F_ST_* (Fig. 1C) as well as the proportion of SNPs where the observed temporal *F_ST_* is greater than expected by chance (Fig. S1B) increases with the difference in time between samples. The temporal *F_ST_* increases non-linearly with duration of time between samples (*slope*_log-log_ = 0.59, *p*_log-log slope = 1_ = 0.0004, *df* = 19). Genome-wide average temporal *F_ST_* appears to asymptote by ∼7 months, corresponding to the duration of time between fall samples and the subsequent spring sample. Remarkably, samples of the Pennsylvanian population collected one to three years apart are as differentiated as populations separated by 5–10° latitude, demonstrating high genetic turnover through time.

### Identification and genomic features of seasonal SNPs

We sought to identify alleles whose frequency consistently and repeatedly oscillated between spring and fall over three years with the assumption that these polymorphisms would be the most likely to be adaptively responding to selection pressures that oscillate between the seasons. We identified seasonally variable polymorphisms that had a large and recurrent deviation from spring to fall around the average frequency using a generalized linear model (GLM) of allele frequency change as a function of season (spring or fall) that took into account read depth and the number of sampled chromosomes (see Materials and Methods for details).

**Figure 2:**
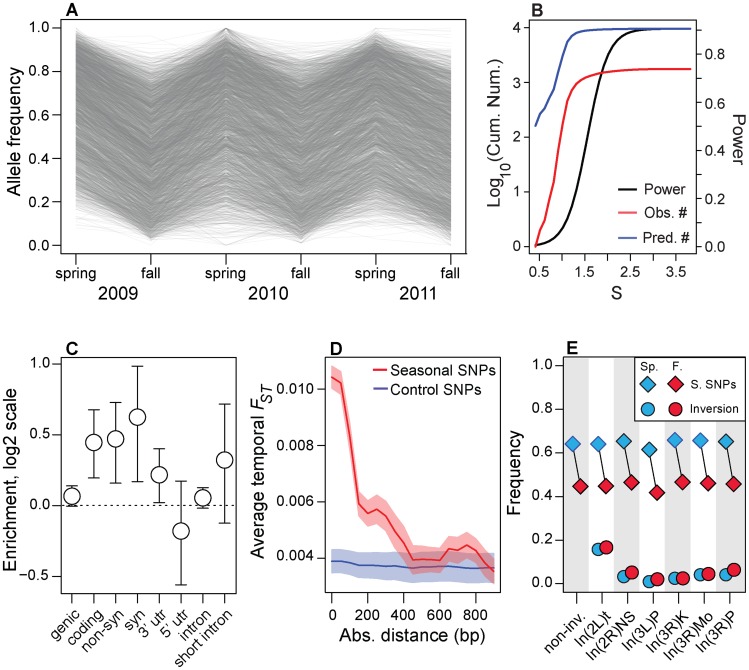
Genomic features of seasonal SNPs. (A) Allele frequency change at each of the ∼1750 seasonal SNPs. Allele frequencies are polarized so that spring allele frequencies are higher than fall allele frequencies. (B) Power to detect seasonal SNPs (black line) is limited and we estimate that we have only identified ∼10% (red line) of all SNPs that repeatedly change in frequency through time (black line). The units of the x-axis (*S*) are the cumulative selection coefficient. See the Materials and Methods for the definition of *S*. (C) Enrichment (log_2_ odds ratio) of seasonal SNPs that are annotated for each class of genetic element relative to control polymorphisms. (D) Seasonal *F_ST_* surrounding seasonal SNPs decays to background levels by ∼500 bp. (E) Allele frequency estimates at seasonal SNPs outside any large, cosmopolitan inversion (non-inv) or within the cosmopolitian inversions (diamonds) during the spring (blue) or fall (red). Allele frequency estimates at SNPs perfectly linked to the inversion during the spring and fall are denoted by circles. Error bars (C) and confidence bands (D) represent 95% confidence intervals based on blocked bootstrap resampling.

Of the ∼500,000 common SNPs tested, we identified approximately 1750 sites that cycle approximately 20% in frequency between spring and fall at FDR less than 0.3 (hereafter ‘seasonal SNPs’; Fig. 2A, Fig. S2A). Statistically significant changes in allele frequency of this magnitude at seasonal SNPs correspond to selection coefficients of 5–50% per locus per generation (Fig. 2B, see Materials and Methods), assuming 10 generations per summer or 1–2 generations per winter. Given the statistical power of our experiment (Fig. 2B), we estimate there may be as many as 10 times as many sites that could cycle either directly in response to seasonally varying selection or could be linked to seasonal SNPs.

Our rationale for focusing on the1750 seasonal SNPs at the FDR of 0.3 is that we are seeking to assess general molecular and evolutionary features of polymorphisms that may underlie rapid adaptive evolution in response to seasonal fluctuations in selection pressure. To assess these general features and enrichments, we require a sufficient number of true positive SNPs while maintaining as low a false positive rate as possible. Reducing FDR rates to lower values yielded an insufficient number of polymorphisms to assess enrichments with adequate precision (FDR of 10% yields 11 SNPs; FDR cutoff of 20% yields 200 SNPs).

We note that our estimation of ∼1750 seasonal SNPs and their associated FDR should only be taken as a rough estimate of the number of seasonally varying SNPs: variance in linkage disequilibrium through the genome, heterscedasticity due to possible demographic events, limited statistical, unbalanced sampling of flies and variance in read-depth among samples, and modeling assumptions will affect our ability to infer the exact number of seasonally varying SNPs. One way to address some of these issues (e.g., heteroscedasticity) is to model allele frequency change through time with generalized linear mixed-effect (GLMM) or general estimation equation (GEE) models that account, to varying degrees, for the structured, time-series nature of our data. Seasonal SNPs inferred with these models are highly congruent with seasonal SNPs inferred using a simple GLM (Fig. S2D,E) and *q-q* plots of the distribution of *p*-values from GLM, GLMM and GEE models suggest that GLM and GLMM modeling strategies fit the bulk of the genome well, with GEE models appearing to be anti-conservative (Fig. S2B,C). However, the identification of a statistical excess of seasonally oscillating SNPs by any modeling strategy will be subject to a number of assumptions that will almost certainly be violated in some way or another and such violations could possibly lead to an increased false-positive rate.

Because the false positive and false negative rates are inherently difficult to estimate, we adopt an empirical strategy to demonstrate that the seasonal SNPs identified though a simple GLM are not a random sample of SNPs but rather are enriched for true positive SNPs that directly underlie the adaptive response to seasonal fluctuations selection pressure. The identified seasonal SNPs are enriched for many signatures consistent with natural selection relative to control SNPs that are matched for several biologically and experimentally relevant parameters such as chromosome, recombination rate, allele frequency, and SNP quality coupled with a rigorous blocked-bootstrap procedure that accounts for the spatial distribution of seasonal SNPs along the chromosome (see Materials and Methods and Table S3). We now proceed to demonstrate these enrichments.

Seasonal SNPs are enriched among functional genetic elements. These polymorphisms are likely to be in genic (i.e., 3′ and 5′ UTR, synonymous and non-synonymous, and long-intron SNPs; *p* = 0.054) and coding regions (synonymous and non-synonymous; *p*<0.002) and are enriched among synonymous (*p*<0.002), non-synonymous (*p* = 0.002) and 3′ UTR (*p* = 0.024, Fig. 2C) relative to control, putatively neutral polymorphisms in short-introns [43]. The *p*-values of the enrichment tests were calculated after controlling for the spatial distribution of seasonal SNPs along the chromosome using a block bootstrap procedure coupled with the identification of paired control SNPs matched for several key genomic features (Table S3), such as recombination rate, average allele frequency in the Pennsylvanian orchard, chromosome, and SNP quality (see ‘Block Bootstrap’ section in Materials and Methods). Enrichment of adaptively oscillating polymorphisms among these genetic elements, including synonymous sites, suggests that these SNPs may affect organismal form and function through modification of protein function, translation rates, or mRNA expression and stability [43], [44].

Next, we show that rapid shifts in allele frequency at seasonal SNPs perturb allele frequencies at nearby SNPs. Adaptively oscillating polymorphisms are in regions of elevated temporal *F_ST_* (Fig. 2D) and the elevation of temporal *F_ST_* decays, on average, by ∼500 bp, consistent with patterns of linkage disequilibrium in *D. melanogaster* [45]. Elevation of temporal *F_ST_* within 500 bp of seasonal SNPs could contribute to high levels of genome-wide average *F_ST_* through time (Fig. 1C). However, excluding SNPs within 500 bp of seasonal SNPs did not change patterns of genome-wide differentiation through time suggesting that genome-wide patterns of *F_ST_* through time are not driven by the seasonal SNPs themselves nor the SNPs in their immediate vicinity (Fig. S3).

**Figure 3:**
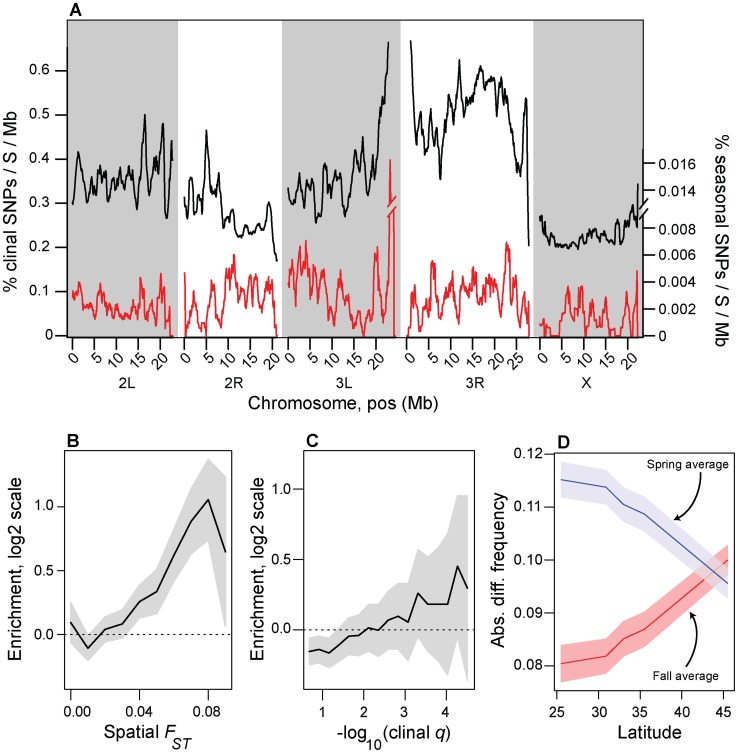
Spatial and temporal variation in allele frequencies. (A) Genomic distribution of clinal (black line) and seasonal SNPs (red line) per megabase per common polymorphism used in this study (Table S1). (B). Enrichment (log_2_ odds ratio) of seasonal SNPs with spatial *F_ST_* greater than or equal to value on *x*-axis relative to control SNPs. (C) Enrichment (log_2_ odds ratio) of seasonal SNPs with –log_10_(spatial q-value) greater than or equal to value on *x*-axis relative to control SNPs. (D) Absolute difference between average spring (blue) and fall (red) frequencies in the Pennsylvanian population and frequency estimates along the cline. Confidence bands represent 95% confidence intervals based on blocked bootstrap resampling.

Seasonal SNPs are spread throughout the genome (Fig. 3A) and there is a 95% chance of finding at least one seasonal SNP per megabase of the euchromatic genome. This result suggests that seasonal SNPs are not exclusively concentrated in any single region (such as an inversion) nor distributed among a small number of regions (such as a limited number of genes). Although seasonal SNPs are distributed throughout the genome, their distribution is over-dispersed. To assess this, we calculated the number of seasonal SNPs per 1000 SNPs under investigation in non-overlapping windows of 1000 SNPs. If seasonal SNPs are homogeneously distributed throughout the genome, the rate of seasonal SNPs/1000 SNPs should follow a Poisson distribution with mean equal to the variance. After accounting for heterogeneity in recombination rate throughout the genome (see Materials and Methods), we find that the variance in the rate of seasonal SNPs is ∼2.3 times greater than expected under a Poisson distribution (*p*<10^−10^) implying that some regions have an excess of seasonal SNPs and some have a deficit of seasonal SNPs. The overdispersion of seasonal SNPs throughout the genome could be caused by several factors including variation in the density of functional elements, multiple functional and clustered seasonal SNPs, variance in the age of seasonal SNPs, or inversion status.

In general, we find no evidence that seasonal SNPs are enriched among large, cosmopolitan inversions segregating in North American populations (*p*>0.05, Fig. S4), with only one inversion, *In3R(Mo)*, marginally enriched for seasonal SNPs (*p* = 0.02, with *p* = 0.18 after Bonferroni correction for multiple testing). In addition, seasonal SNPs are significantly more common in the Pennsylvanian orchard population than polymorphisms perfectly linked [46] to large cosmopolitan inversions (Fig. 2E) and polymorphisms linked to inversions do not vary between seasons (Fig. 2E, *p*>0.05), including those linked to *In3R(Mo)*. Therefore, enrichment of seasonal SNPs within *In3R(Mo)*, if present, is most likely due to increased linkage disequilibrium caused by decreased recombination surrounding this inversion [47]. Taken together, these results indicate that the inversions themselves do not cycle seasonally in the Pennsylvanian population in any appreciable manner (Fig. 2E) and suggests that adaptive evolution to seasonal variation in selection pressures may be highly polygenic.

### Relationship between spatial and temporal variation in allele frequencies

To test the hypothesis that spatially varying selection pressures along the latitudinal cline reflect seasonally varying selection pressures in the Pennsylvanian population, we examined the relationship between temporal and spatial variation in allele frequencies. To quantify spatial variation in allele frequency, we calculated two statistics. First, we estimated average pairwise *F_ST_* among all populations for each SNP (‘spatial *F_ST_*’). Second, we estimated clinality for each SNP by calculating the per-SNP false discovery rate (FDR) of the relationship between allele frequency and latitude using a generalized linear model that takes into account read depth and the number of sampled chromosomes (hereafter ‘clinal *q*-value’). Spatial *F_ST_* and clinal *q*-value are highly correlated (*r* = 0.63, *p*<1e-10; Fig. S5) demonstrating that most, but not all, spatial variation along the latitudinal cline is represented by monotonic changes in allele frequency between northern and southern populations.

We calculated the number of clinally varying polymorphisms (clinal *q*-value<0.1) and the number of adaptively oscillating polymorphisms per common segregating SNP (average, North American MAF>0.15) per megabase of the genome (Fig. 3A). Approximately one out of every three common polymorphisms varies with latitude with FDR<0.1 (i.e., clinal *q*-value<0.1) whereas only one out of every three thousand polymorphisms varies predictably between seasons with seasonal FDR<0.3 (Fig. 3A). Although our ability to detect clinal SNPs at FDR<0.1 is greater than our ability to detect seasonal SNPs at FDR<0.3 (cf. Fig. 2B, Fig. S6), differences in power cannot explain the three order of magnitude difference in the number of detected clinal and seasonal SNPs (cf. Fig. 2B, Fig. S6).

Next, we formally tested whether seasonal SNPs are enriched among spatially varying SNPs. Spatially varying SNPs, as defined by spatial *F_ST_*, are more likely to be seasonal SNPs than expected by chance (Fig. 3B), and the odds of this enrichment increases with increasing spatial differentiation. In contrast, we cannot reject the null hypothesis of no enrichment of seasonal SNPs among clinal SNPs as defined by clinal *q*-value (Fig. 3C).

The observed differences in the enrichment of seasonal SNPs among SNPs with high spatial *F_ST_* and low clinal *q*-value may reflect aspects of our sampling design and differences in the evolutionary forces that shape allele frequencies through time and space. We sampled flies along the East Coast during different years and at different points of time relative to the progression of the growing season in each population (Table S1). Thus, in each sampled clinal population, seasonal SNPs would be at different points in their adaptive trajectory. Consequently, seasonal SNPs would not likely have exceedingly low clinal *q*-values, a statistic which reflects the deviation of observed allele frequencies from the predicted value as estimated by a GLM. Rather, seasonal SNPs would likely be highly differentiated along the cline (i.e., have a large spatial *F_ST_*). SNPs with low clinal *q*-values, therefore, represent those SNPs that do not change in frequency between seasons and possibly reflect long-term demographic processes or adaptation to selection pressures that vary clinally, but not seasonally.

Because of the relationship between spatial differentiation and seasonal variation in allele frequencies (Fig. 3B) and because of parallels between spatial and seasonal variation in climate, we hypothesized that northern populations should be more ‘spring-like’ and southern populations should be more ‘fall-like’ in allele frequencies at the seasonal SNPs. To test this hypothesis, we calculated the absolute difference in allele frequencies for each population sampled along the cline with the average spring and fall allele frequency estimates for the Pennsylvanian population for all seasonal SNPs. Indeed, allele frequency estimates at seasonal SNPs from high latitude populations are more similar to spring Pennsylvanian populations and those from low latitude are more similar to fall populations (Fig. 3D) demonstrating that latitudinally varying selection pressures at least partially reflect seasonally varying selection pressures.

### Immediate adaptive response to an acute frost event

**Figure 4:**
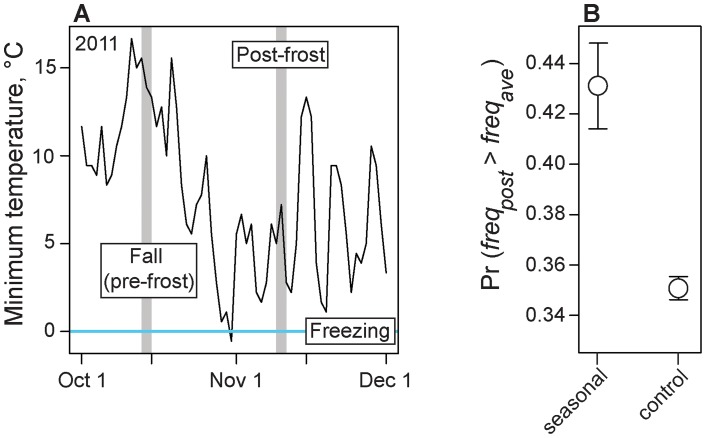
Adaptive evolution to frost. (A) Temperature records at a weather station close to the focal orchard. Grey lines indicate collection dates for pre- and post-frost samples. (B) Probability that post-frost allele frequencies at seasonal and control SNPs overshoot the long-term average (based on 2009 and 2010 estimates) allele frequency at each site. Confidence intervals based on blocked bootstrap resampling.

In the late fall of 2011, about two weeks after our 2011 fall sample was collected, a hard frost occurred in the Pennsylvanian orchard (Fig. 4A). We were able to obtain a sample of *D. melanogaster* approximately one week after the frost and we estimated allele frequencies genome-wide from this sample. We hypothesized that allele frequencies at seasonal SNPs would predictably change following the frost event and would become more ‘spring-like.’ To test this hypothesis, we calculated the probability that post-frost allele frequencies at seasonal SNPs overshoot the long-term average allele frequency (i.e., become more ‘spring-like’). We also estimated this probability for control polymorphisms, matched to adaptively oscillating polymorphisms by several characteristics (Table S3) including, importantly, difference in allele frequency between the long-term average and the pre-frost allele frequency. This later control is essential given that some shift in the ‘spring-like’ direction is expected here simply by chance due to regression to the mean. The probability that seasonal SNPs overshoot the long-term average allele frequency is ∼43%, whereas only ∼35% of control polymorphisms overshoot the long-term average. This significant excess of adaptively oscillating polymorphisms that become more ‘spring-like’ following the frost event (Fig. 4B; log_2_(OR) = 0.48, *p*<0.002) suggests that these SNPs respond to acute changes in climate and that cold temperatures associated with winter is one selective force acting on this population shaping allele frequencies between seasons.

### Association with seasonally variable phenotypes

**Figure 5:**
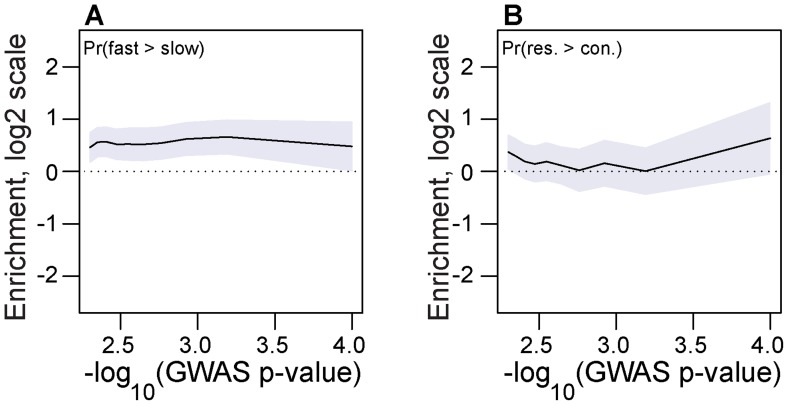
Association with seasonally variable phenotypes. Enrichment (log_2_ odds ratio) of seasonal SNPs that change in frequency in the expected direction at SNPs associated with chill coma recovery time (A) and starvation tolerance (B) relative to contronl SNPs. The x-axis represents the threshold -log_10_(GWAS *p*-value), i.e. values along the x-axis represent the minimum -log_10_(GWAS *p*-value) for SNPs under consideration. Error bars represent 95% confidence intervals based on blocked bootstrap resampling.

Chill-coma recovery time and starvation tolerance are two phenotypes that vary seasonally in drosophilid populations [48]–[53]. Accordingly, we hypothesized that the winter-favored allele at seasonal SNPs would be associated with decreased chill-coma recovery time and increased starvation tolerance. To test this hypothesis, we used allele frequency data from previously published tail-based mapping of chill-coma recovery time and starvation tolerance [54]. We show that the winter favored allele at seasonal SNPs is more likely to be associated with fast chill coma recovery time than expected by chance across a range of GWAS *p*-values (Fig. 5A). A similar analysis of starvation tolerance was equivocal but the general pattern is that the winter-adaptive allele is associated with increased starvation tolerance (Fig. 5B).

### Long-term balancing selection

Balancing selection caused by variation in selection pressures through time can in principle maintain allelic variation at adaptively oscillating loci and elevate levels of neutral diversity surrounding these balanced polymorphisms. Thus, if seasonal variation in selection pressures promotes balanced polymorphisms we hypothesized that seasonal SNPs would be old and present in regions of elevated polymorphism.

We tested the hypothesis that seasonal SNPs are old by first examining their allele frequencies in a broad survey of African *D. melanogaster* populations [55]. Approximately 5% of seasonal SNPs are rare in Africa (MAF<0.01), however these SNPs are not more likely to be rare in Africa than control polymorphisms (log_2_(odds ratio) = 0.96; *p* = 0.328). Interestingly, for seasonal SNPs where one allele is rare in Africa, the summer favored alleles are more likely to be rare in Africa than winter favored alleles (log_2_(odds ratio) = 0.475; *p* = 0.018). Because the vast majority of seasonal SNPs segregate in Africa, it appears that adaptation to temperate environments, and particularly winter conditions, relies primarily on old, standing genetic variation.

Balancing selection acts to maintain alleles at intermediate frequencies for long periods of time and, in some instances, can maintain polymorphism across species boundaries [56], [57]. We examined whether seasonal SNPs showed signatures of long-term balancing selection by examining patterns of polymorphism surrounding orthologous regions in *D. simulans*, the sister species to *D. melanogaster*. We note that the following analyses are conservative because we underestimate *D. simulans* diversity given the small number (<6) of *D. simulans* haplotypes used.

**Figure 6:**
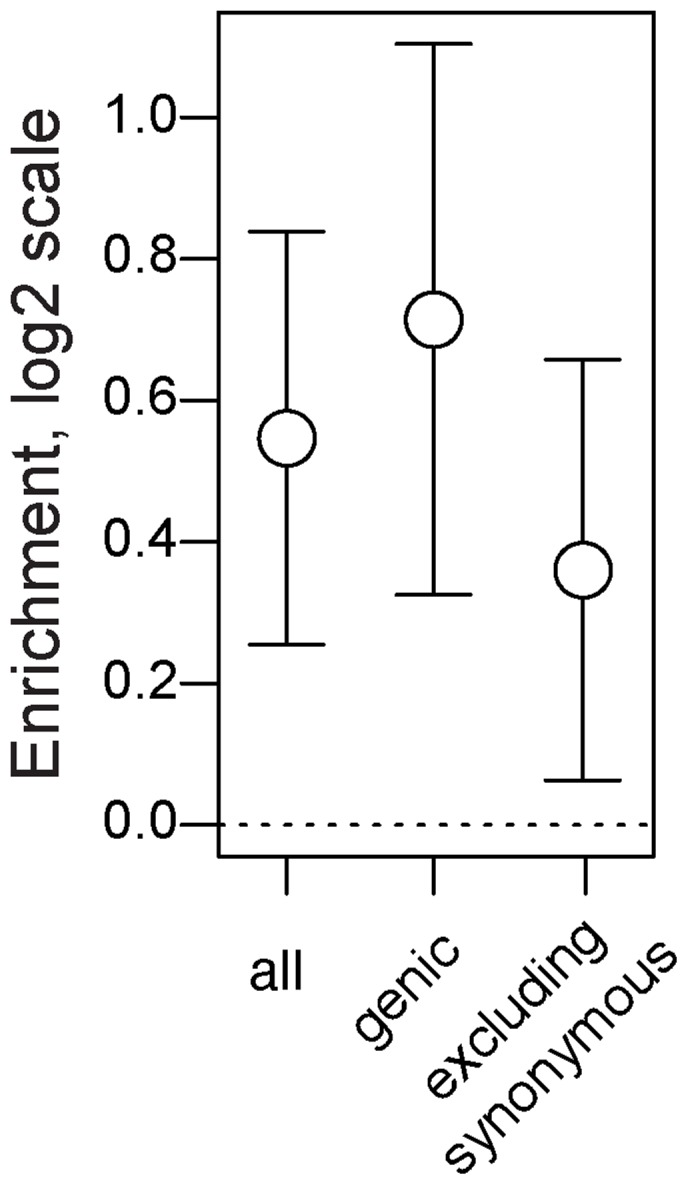
Long term balancing selection. Enrichment (log_2_ odds ratio) of seasonal SNPs among SNPs that polymorphic and identical by state among 6 lineages of *D. simulans* relative to control SNPs. Error bars represent 95% confidence intervals based on blocked bootstrap resampling.

First, we demonstrate that seasonal SNPs are approximately 1.5 times more likely to be polymorphic and share the same two alleles identical by state in both species relative to control SNPs. This pattern is observed for all seasonal SNPs (Fig. 6, *p*<0.002) and for seasonal SNPs residing in genes (Fig. 6, *p*<0.002). The increased probability of shared polymorphism between *D. melanogaster* and *D. simulans* at seasonal SNPs could, in principle, be driven by an over-representation of synonymous, genic SNPs (Fig. 2C). Unless synonymous SNPs are in four-fold degenerate positions, certain mutations may cause them to be non-synonymous thereby limiting the number of possible neutral allelic states and increasing the probability of shared polymorphism between species. However, adaptively oscillating SNPs that do not reside in synonymous sites are also more likely than expected by chance to be polymorphic and share the same two alleles by state in *D. melanogaster* and *D. simulans* (Fig. 6, *p* = 0.014).

The co-occurrence of shared polymorphism between *D. melanogaster* and *D. simulans* could result from three evolutionary mechanisms. First, trans-specific polymorphisms could result from adaptive introgression. This scenario seems implausible given the high degree of pre- and post-zygotic isolating mechanisms between these two species [58], [59]. Furthermore, if trans-specific polymorphisms resulted from recent adaptive introgression we would expect average pairwise divergence between *D. melanogaster* and *D. simulans* surrounding seasonal SNPs to be smaller than at control SNPs. However, there is no significant difference in estimates of divergence between seasonal and control SNPs (*p* = 0.7 for windows ±250 bp). Second, trans-specific polymorphisms could result from convergent adaptive evolution. Finally, trans-specific polymorphisms could be millions of years old [60], predating the divergence of *D. melanogaster* from *D. simulans*. While we cannot differentiate these latter two mechanisms, we postulate that the most parsimonious explanation is that trans-specific seasonal SNPs predate the divergence of these two sister species.

### Seasonally variable selection is required to generate genome-wide patterns of allele frequency change through time

Despite empirical support for the conclusion that seasonal SNPs show many signatures consistent with adaptive response to seasonally variable selection, drift, caused by cyclic population booms and busts, or migration from neighboring demes are alternative mechanisms that could drastically perturb allele frequencies in the Pennsylvanian population and could generate some of the genome-wide patterns we observe. We address these possibilities here and conclude that neither cyclic changes in population size nor seasonal migration can plausibly explain the extent of genome-wide genetic differentiation through time, the observed number of seasonal SNPs, nor the enrichment of seasonal SNPs among many distinct genomic features (e.g., Figs. 2–6). At the same time, we also show through several simulation approaches that rapid adaptive evolution in response to seasonal fluctuations in selection pressure is sufficient to explain patterns of allele frequency change through time. Furthermore, we discuss how large-scale migration is internally inconsistent with certain aspects of our data. Taken together, we conclude that rapid adaptive evolution to seasonally variable selection is required to explain the patterns of allele frequency change through time at seasonal SNPs and at linked neutral loci that we observe in our dataset.

First, we assessed the possibility that extensive drift caused by population contraction every winter [31], [61], [62] could generate genome-wide patterns of genetic differentiation through time observed in our data. To do so, we conducted forward genetic simulations that model biologically plausible variation in population size and included loci that cycle in frequency due to variable selection pressures [63]. For these simulations, we modeled a 20 Mb chromosome with constant recombination rate of 2 cM/Mb, representing the genome-wide average recombination rate in *D. melanogaster* [64]. We simulated population contraction to one of various minimum, ‘overwintering’ population sizes followed by exponential growth over 10 generations in the ‘summer’ to a fixed maximum population size. In these models, we included various numbers of loci that respond to seasonally varying selection. Selection coefficients for each locus were set such that allele frequencies at selected sites oscillated by ∼20%, between 60 and 40%, representing the average change in allele frequency we actually see between spring and fall at seasonal SNPs. Finally, we placed 500 neutral loci randomly along the simulated chromosome and measured *F_ST_* at these neutral loci between three ‘spring’ (i.e., first generation of population expansion) and ‘fall’ (last generation of population expansion) samples. See Materials and Methods for more details these models.

**Figure 7:**
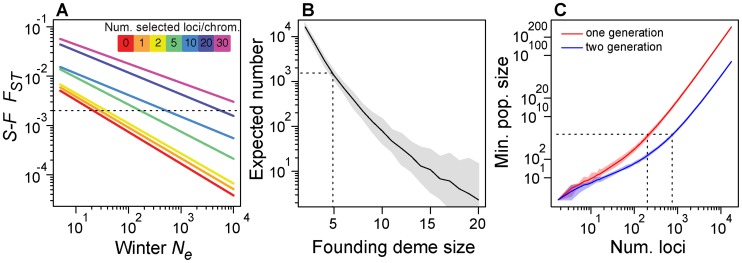
Demographic models. (A) Expected value of *F_ST_* between simulated spring and fall samples (y-axis), conditional on overwintering effective population size and the number of seasonally adaptive alleles (color key). Dotted line represents observed average, genome-wide after *F_ST_* between spring and fall samples from the Pennsylvanian population. (B) Expected number of SNPs that would vary repeatedly between seasons three times in a row conditional on founding deme size for a simple model of recolonization of the orchard population. Dotted line represents the observed number of seasonal SNPs and the corresponding founding deme size required, in this case 5 flies. (C) Minimum population size (y-axis) for the required for varying number of seasonally selected loci (x-axis) under a truncation selection model assuming independent response to selection at each locus. Dotted line represents our best guess of fall population size and corresponding number of loci that could independently respond to truncation selection. Confidence bands based on resampling of observed allele frequency change at seasonal SNPs.

In the absence of seasonal selection, these forward simulations suggest that overwintering *N_e_* would have to be exceedingly low (∼20; Fig. 7A) to generate levels of *F_ST_* between spring and fall as high as we observe in our data (arrow in Fig. 1C). However, with overwintering *N_e_* of 200 and 5–10 seasonally adaptive SNPs per chromosome arm, simulated *F_ST_* at neutral loci is on the order of 0.002 (Fig. 7A), which we observe in our data (arrow in Fig. 1C). While we do not know overwintering population size, we speculate it could be on the order of 200 flies or likely substantially larger [61], [62] and conclude that at least 25–50 (5–10 per main chromosome arm) loci are sufficient to generate patterns of differentiation we observe through time. Note that increasing the overwintering population size requires concomitant increase in number of seasonally selected loci.

We regard overwintering population sizes of ∼20 flies to be inconsistent with certain aspects of our data and also implausible given what we know about the biology of the species. First, such a severe population contraction would result in reduction of genetic diversity, particularly for low frequency alleles. However, the observed allele frequency spectrum between fall and the following spring samples is similar and spring samples do not exhibit the expected loss of low frequency polymorphisms that would result from a population contraction to 20 individuals (Fig. S7). Second, population contraction to 20 individuals would often lead to population extirpation in the Pennsylvanian orchard and would certainly lead to extirpation at localities further north that experience more severe winters. However, *D. melanogaster* are routinely collected in Northern orchards very early in the season [65] and are routinely found in populations at as far north as 45° (Schmidt pers. obs). Furthermore, certain rare alleles have persisted in northern *D. melanogaster* populations for upwards of 30 years [66] cf. [67] and allele frequency clines are relatively stable over decadal scales [68] demonstrating that high latitude populations are not frequently extirpated and that overwintering bottlenecks cannot be so severe as our neutral simulations would require.

In our forward simulations, seasonally variable selection is sufficient to generate high levels of genome-wide genetic differentiation through time. In addition, our forward simulations are consistent with the increase of genome-wide average *F_ST_* through time excluding polymorphisms that are within 500 bp of seasonal SNPs (Fig. S3). In our simulations, 500 neutral loci were placed randomly along a 20 Mb chromosome and were initially completely unlinked to selected loci. Therefore, the high levels of simulated *F_ST_* are a consequence of genetic draft acting over long physical distances with low to moderate linkage disequilibrium between neutral and selected polymorphisms. Our observation that genome-wide average *F_ST_* (excluding polymorphisms near seasonal SNPs, Fig. S3) increases with time resembles our simulations suggesting that draft can perturb allele frequencies over long genetic distances.

We also note that long-range genetic draft, caused by rapid frequency shifts of ancient balanced alleles to seasonally variable selection would likely cause an asymptotic change in genome-wide temporal *F_ST_*, whereas a purely drift-based model would likely cause a linear increase in genome-wide *F_ST_* through time. Seasonal SNPs tend to be old and are therefore likely found on a diverse array of haplotypes. Therefore, the exact composition of haplotypes that rise and fall every seasonal cycle will be somewhat stochastic giving rise to a high genome-wide *F_ST_* over a duration of time less than ∼7 months (the duration of time between fall and the following spring). Among years, genome-wide average *F_ST_* would possibly plateau if local *N_e_* were large (as we suspect it is, see Results and Discussion: The plausibility…), coupled with the effects of recombination, gene conversion, and low-level migration from neighboring demes or populations. Finally, we note that because seasonal SNPs likely exist on a diverse array of haplotypes we do not expect genome-wide average *F_ST_* to oscillate with a period corresponding to approximately 6–7 months. For such oscillations to occur, a large (i.e., much larger than we identify) number of loci would have to be repeatedly shifting between seasons.

Next, we explore the possibility that migration could drastically alter allele frequencies in the Pennsylvanian population and generate the large number of loci that vary repeatedly among seasons. First, we examined a simple but general demographic model where the Pennsylvanian orchard population becomes extirpated every year and recolonized from a refugium such as a southern population or a large, local site such as a compost pile. Either situation is plausible given the purportedly high rates of migration in North American *D. melanogaster* populations [67], [69] and what little is known about the overwintering biology of high latitude *D. melanogaster* [66]. In our model, we envisioned a resident, refugial population with stable allele frequencies across years that colonizes the orchard population. In this model, the orchard would be colonized early in the season with a random subsample of flies from the refugium and would therefore have aberrant allele frequencies. As more migrants arrived to the orchard from the refugium, allele frequencies at the orchard would stabilize to that of the source population. In such a scenario, allele frequencies in spring samples could vary considerably and a small fraction of SNPs might, by chance, have the same aberrant allele frequencies year after year and would appear to cycle seasonally.

We calculated the expected number of SNPs that would cycle by chance alone as a function of the number of initial migrants (Fig. 7B). For instance, if five migrants arrived at the orchard prior to our spring sample every year, approximately 1300 SNPs would cycle seasonally producing similar patterns to the observed change in allele frequency through time as at ‘seasonal SNPs’ (Fig. 2A). However, if four migrants arrived at the orchard prior to our sampling, ∼2600 SNPs would vary repeatedly but if six migrants arrived, only ∼700 would. Although the expected number of sites that oscillate under this migration model with 5 migrants is approximately the number we observe, we note that the expected number is highly dependent on the exact number of migrants. It seems unlikely that exactly five flies would migrate from the refugium to the orchard before our first spring sample three times in a row. Therefore, the extreme sensitivity of the expected number of sites to the number of migrants makes this general demographic scenario implausible. We are therefore led to conclude that the simple migration model presented here is likely to be insufficient to explain changes in allele frequency through time in the Pennsylvanian orchard.

In addition to our conclusion that a simple model of recolonization of the orchard is insufficient to explain the number of seasonally variable loci we observe, our data are also inconsistent with large-scale migrations from adjacent populations. For instance, if a large-scale migration from the South to resident northern populations were to occur, we would expect that clinally varying SNPs should also vary seasonally. Such a pattern would be expected both if a large-scale migration occurred randomly or were genotype dependent. However, seasonal SNPs are apparently not enriched among clinally varying polymorphisms (Fig. 3C). A similar logic would apply for an early season migration from the North followed by a subsequent, late season migration from the South. We also note that this dual migration model is biologically implausible. The relationship between latitude and the onset of spring would suggest that far northern populations would be quite small in the early part of the growing season and the subsequent probability of emigration to southern locales would be low. Therefore, we conclude that large-scale migration does not play a major role shaping seasonal variation in allele frequencies in the Pennsylvanian orchard. Furthermore, even if seasonal SNPs were enriched among clinally varying polymorphisms (which they do not appear to be), adaptation to seasonally variable selection would need to be invoked in order to explain the yearly shift in allele frequencies every winter.

Taken together, the models presented here demonstrate that seasonal boom-bust or migration-based scenarios are insufficient to explain allele frequency change through time in the Pennsylvanian population. While temperate populations of *D. melanogaster* clearly undergo cyclic population booms and busts due to changes in climate associated with the season, the extent of these population contractions necessary to generate the patterns of genetic variation through time that we observe would be too extreme to allow for stable population persistence. Similarly, the Pennsylvanian population certainly exists as a part of a complex metapopulation and experiences immigration and emigration. However, analysis of a simple demographic model of population recolonization during the spring is also insufficient to explain the patterns of allele frequency change through time that we observe and our data are internally inconsistent with a model of large-scale migration from neighboring populations.

Finally, we point out that the boom-bust and recolonization models we presented here undoubtedly are oversimplifications and that there are other, more complex demographic models that we have not explored. Nonetheless, any stochastic demographic event would affect SNPs throughout the genome with equal probability. Many aspects of our data clearly show that seasonal SNPs are not a random set of common SNPs but rather show signatures consistent with both functional effect and long-term balancing selection such as enrichment in specific classes of genetic elements, association with seasonally variable phenotypes and predictable and virtually instantaneous shifts in allele frequency in response to frost. Therefore, while we cannot conclusively rule out the possibility that demographic events affect the temporal dynamics of allele frequencies at seasonal- and non-seasonal SNPs in the Pennsylvanian population, these demographic events are most likely coupled with adaptive evolution in response to temporally varying selection pressures.

### The plausibility of seasonally variable selection

We have previously argued that adaptive response to seasonally fluctuating selection at no less than 25–50 loci is necessary to generate the high levels of genome-wide genetic differentiation through time observed in the Pennsylvanian population. Next, we considered the plausibility of such strong selection and estimated the upper bound of the number of loci that could independently respond to seasonally variable selection. To do so, we modeled independent selection at 1–10,000 simulated seasonal SNPs whose allele frequency change was drawn from the observed allele frequency change at seasonal SNPs. Using a simple Poisson model (see Materials and Methods), we estimated the minimum fall census size required for that number of loci to shift in allele frequency during one or two rounds of truncation selection. Using these models, we sought to estimate the most likely number of seasonal SNPs that could independently respond to seasonally variable selection by contrasting model-based estimates of population size with our best estimates of population size in the field.

Although fall census size of *D. melanogaster* in the focal Pennsylvanian population is unknown, some estimates of drosophilid population size have been made. Global population size of *D. melanogaster* is likely to be extremely large, greater than 10^8^ [70]. However, estimates of local population size made from mark-release-recapture methods report census sizes on the order of 10^4^ to 10^5^ [71]–[73]), with considerable variation among seasons, years and locales. *D. melanogaster* samples from orchards and vineyards often exceed 10^4^ flies [74], [75] and thousands of flies can easily be collected over large compost piles (Bergland pers. obs.). Therefore, we speculate that census size of temperate *D. melanogaster* populations at any locale is a function of the local ecology (e.g., amount of windfall fruit, number and size of compost piles, humidity) and given the favorable conditions in the focal Pennsylvanian orchard (Schmidt pers. obs.), large census sizes of more than 10^5^ are conceivable. If fall census size in the Pennsylvanian population is on the order of 10^5^, our truncation selection model suggests that no more than several hundred (200–700, Fig. 7C) seasonal SNPs could respond to seasonally varying selection independently. We note that increasing the number of generations of winter-like selection pressures or the fall census size would lead to a concomitant increase in the number of seasonally selected loci that could independently respond to seasonally varying selection pressures.

Our survey of temporal changes in allele frequency identified 1750 seasonal SNPs that cycle significantly by ∼20% between seasons at FDR of 0.3. Unless local census size in the Pennsylvanian population were unrealistically large – on the order of 10^10^ or 10^20^ – it is unlikely that all of these loci respond to selection independently. Our model suggests, however, that a large fraction, on the order of 200–700 could vary independently in every cycle. One explanation for cycling in the remaining SNPs is linkage with loci responding to seasonally variable selection. It is possible that this linkage is generated either stochastically and neutrally or, alternatively, by selective processes such as assortative mating [76] or epistatic selection [77], [78]. For instance, if winter adapted flies were more likely to mate with other winter adapted flies during the summer, winter adapted alleles may become coupled and linkage disequilibrium between these alleles could increase. Similarly, certain forms of epistatic interactions could also generate linkage disequilibrium between seasonal SNPs if, for instance, couplings of winter and summer favored alleles at multiple loci were particularly deleterious relative to winter-winter or summer-summer combinations. The net effect of selective mechanisms that promote positive linkage disequilibrium between seasonal SNPs is that the effective number of ‘independently’ seasonally selected loci decreases. If seasonal SNPs are in linkage disequilibrium due to selective processes, it would imply that more than 200–700 seasonal SNPs contribute to organismal form and function and modify fitness during the summer and winter.

### Conclusions – Summary

Herein, we present results from population based resequencing of samples of flies collected along a latitudinal cline in North America and over three years during the spring and fall in a Pennsylvanian orchard. We identify repeatable and dramatic changes in allele frequencies through time at hundreds of polymorphisms spread throughout the genome. Response to strong selection at these seasonal SNPs likely drives genetic differentiation through time at linked, neutral polymorphisms. This process leads to genome-wide differentiation between samples collected several years apart comparable to populations separated by 5–10° latitude. Seasonal SNPs are likely to be functional as they show enrichment at functional sites, vary predictably among populations sampled along the cline, respond immediately to a hard frost event, and are associated with phenotypes previously shown to vary seasonally in temperate *D. melanogaster* populations. Finally, our results suggest that some adaptively oscillating SNPs are possibly millions of years old, predating the split of *D. melanogaster* from its sister species *D. simulans*. Taken together, our results provide the first genomic picture of balancing selection caused by temporal fluctuations in selection pressures and provide novel insight into the biology of marginal overdominance.

### Conclusions – Functional properties of adaptively oscillating polymorphisms

Temperate populations of *D. melanogaster* are exposed to high levels of environmental heterogeneity among seasons due to changes in various aspects of the environment including temperature, humidity, and nutritional quality and quantity. These shifts in the environment are primary determinants of cyclic population booms and busts [66], [71], [72] and impose strong temporally and spatially variable selection. Intuition, theoretical models [79], laboratory experimentation [35], and inference from patterns of clinal variation [80]–[82] and seasonal variation in morphological, behavioral and life-history traits suggest that alternate seasons favor differing life-history strategies. In general, populations exposed to more harsh conditions such as those from Northern locales or those collected early in the season are larger [83], [84], more stress tolerant [49]–[51], [82], longer lived [81], and are less fecund [81], [85] than those collected in Southern locales or during the fall. The general picture that emerges, therefore, is that in temperate populations winter conditions select for hardier but less fecund individuals whereas summer selects for high reproductive output at the cost of somatic maintenance. Nonetheless, there is surprisingly little evidence directly linking adaptive differentiation between seasonally favored genetic polymorphisms, phenotypes and environmental perturbations (but see [35]). Herein we present several key results that link seasonal and spatial patterns of genotypic and phenotypic variation with environmental perturbations.

First, our data suggest that that acute bouts of cold temperature elicit adaptive response at seasonally oscillating polymorphisms (Fig. 4). Heretofore, the specific environmental factors altering allele frequencies through time and space among dipteran species has generally remained elusive largely stemming from the fact that many aspects of the environment co-vary over temporal and spatial scales. Here we show that acute exposure to sub-freezing temperatures in the field shifts allele frequencies in a spring like direction at seasonal SNPs but not at control polymorphisms, thereby suggesting that sharp modulation of temperature can act as a selective force in the field. While post-frost allele frequencies at seasonal SNPs move in a ‘spring-like’ direction, they do not reach average spring allele frequencies. This suggests that multiple frost events, long-term exposure to cold temperatures or other selective factors linked to winter conditions such as starvation also impose strong selection in temperate populations.

Next, we demonstrate that environmental differences among populations predict, to a certain extent, changes in allele frequency at seasonal SNPs. Environmental factors that vary over seasonal time scales also vary with latitude. This fact has facilitated studies that substitute space for time and has led to a paradigm in many aspects of contemporary research in drosophilid evolutionary ecology of examining phenotypic and genetic differentiation along latitudinal (and altitudinal) clines as a proxy for studying adaptation to temperate environments [e.g., 86]. Using allele frequency estimates that we made from populations sampled along the North American latitudinal cline, we demonstrate that southern populations are more ‘fall-like’ at seasonal SNPs whereas northern populations are more ‘spring-like’ (Fig. 3D). Northern populations experience more severe winters and have shorter growing seasons; therefore, we speculate that the changes in allele frequency at adaptively oscillating polymorphisms along the cline is because (1) the summer favored allele would be at lower frequency due to stronger selection during the winter and (2) the summer favored allele would not rise in frequency as much during the summer because of the shorter growing season. The converse would be the case for Southern populations.

Finally, we relate seasonally variable SNPs with ecologically relevant phenotypic variation. Previous studies have demonstrated that two important stress tolerance traits, chill coma recovery time and starvation resistance vary in predictable ways among temperate populations of *D. melanogaster*. Northern populations tend to have fast chill coma recovery time [87]–[89] recapitulating deeper phylogenetic patterns among drosophilids originating from temperate and tropical locales [48]. Evidence for latitudinal variation in starvation tolerance is more equivocal with low latitude populations of *D. melanogaster* being more starvation tolerant in some studies but not significantly so in others [49], [91] and closely related species showing equally ambiguous patterns [52], [90], [91]. However, diapause-competent genotypes that are at high frequency in Northern populations and in the spring show increased starvation tolerance [52] suggesting that spatial and temporal differentiation in starvation tolerance may be parallel in the context of specific polymorphisms. Nonetheless, because selection pressures along latitudinal clines are generally parallel with seasonal selection pressures (e.g., Fig. 3D) we reasoned that winter adapted alleles at seasonal SNPs would be associated with fast chill coma recovery time and increased starvation tolerance.

We show that winter adapted alleles at seasonal SNPs are likely to be associated with fast chill coma recovery time and, to a lesser extent, starvation tolerance (Fig. 5). The strength of the relationship between seasonal SNPs with these two phenotypes likely differs for many reasons, including intrinsic differences in the statistical power and the complex genetic architecture of these traits. Nonetheless, the fact that seasonal SNPs are associated with chill coma recovery and starvation tolerance in the predicted direction given our prior knowledge of seasonal variation in these two traits strongly suggests that seasonal SNPs are functional and affect seasonally dependent fitness via stress tolerance traits. In addition, the concordance between seasonal SNPs and SNPs moderately associated with chill coma recovery time and starvation tolerance suggests that the intermediate frequency SNPs that we are investigating here have small effects on phenotype but nonetheless have large effects on average population fitness.

Taken together, our analysis has linked adaptive oscillations at hundreds of polymorphisms in *D. melanogaster* to specific and persistent differences in climate and to phenotypes known to be under diversifying selection through time and space. Our results support the hypothesis that stress tolerance traits are favored during the winter and disfavored during the summer. Stress tolerance traits such as chill coma recovery time and starvation tolerance often have negative genetic correlations with reproductive output [52], [92] or development time [93], two phenotypes that would be favored during exponential growth during the summer. Therefore, it is likely that a subset of seasonal SNPs directly contribute to a tradeoff between stress tolerance and reproductive output.

Because *D. melanogaster* originated in sub-Saharan Africa and colonized the world in the wake of human migration 200–10,000 years ago [94] it has been hypothesized [95] that phenotypes favored during the winter are derived whereas those favored during the summer are ancestral with respect to tropical, African populations. Although we show that the vast majority of seasonal SNPs are common in Africa, a small set (∼5%) are rare, segregating at less than 1%. Somewhat surprisingly, summer favored alleles are more likely to be rare in Africa than winter favored alleles (see Results and Discussion: Long term…) suggesting that some environmental aspects of summer in temperate orchards are new for *D. melanogaster*. Consistent with the observation that flies sampled at low latitudes are likely subject to intense intra- and inter-specific competition [83], we speculate that the cornucopia of rotten fruit during the summer in mid- to high-latitude locales coupled with decreased inter-specific competition is a novel environment for *D. melanogaster* that has allowed formerly rare alleles associated with increased reproductive output to flourish.

### Conclusions – Long-term, polygenic balancing selection, and ecological generality

Herein, we present several lines of evidence demonstrating that hundreds of loci adaptively respond to seasonal fluctuations in the environment. Despite (or because of) the fact that these loci promote rapid adaptive evolution, many have remained polymorphic for millions of generations within *D. melanogaster* and some possibly predate the divergence of *D. melanogaster* and *D. simulans* ∼5 million years ago. Taken together, these observations suggest that alleles at these loci have may have been maintained by environmental heterogeneity for exceptionally long periods of time. Long-term balancing selection is typically regarded as an evolutionary oddity, found predominantly in the genetic systems regulating host-pathogen interactions, self-incompatibility, and sex-determination [56], [96]. Herein, we provide evidence that environmental heterogeneity might promote long-term balanced polymorphisms at hundreds of loci that affect quantitative, stress tolerance traits.

Theory predicts that temporal variation in selection coefficients can maintain adaptive genetic variation for long periods of time when certain genetic and ecological conditions are met. Classic models suggest that the adaptive variation can be maintained in populations because of temporal shifts in selection pressure only when the heterozygote has a higher geometric mean fitness than either homozygote [1]. Such conditions are necessary for both finite and infinite populations and, moreover, in finite populations the persistence time of adaptive polymorphisms may be shorter than for neutral ones [8]. However, alternative models have demonstrated that overlapping generations [97], the combination of spatial and temporal variation in selection pressures [4], habitat fidelity [98], [99], and multiple liked loci subject to temporally variable selection [3] will increase the persistence time of balanced polymorphisms maintained by environmental heterogeneity.

Each of these conditions are met in for *D. melanogaster*. First, flies are highly fecund [100], iteroparous insects with generation time a fraction of lifespan [80], [81]. Therefore natural populations are likely to be highly age structured which will prevent the loss of balanced alleles during alternate seasons. Second, spatial selection pressures vary on the order of meters to kilometers [101], [102], all well within the dispersal radius of flies [72]. In addition, flies often return to the substrate they were collected on [103], [104] and flies collected within a locale show signatures of population structure on the order of tens of meters [105], [106]. Therefore, low to moderate levels of migration between demes separated by various distances [67], [69], [72] and environmental heterogeneity over small spatial scales may help mitigate the loss of balanced polymorphisms in any one orchard. Finally, our study identified hundreds of adaptively oscillating polymorphisms. Although the vast majority of these polymorphisms are unlinked due to the large physical distance between them, there is evidence of heterogeneity in the abundance of seasonal SNPs throughout the genome suggesting that some might be in partial linkage disequilibrium. Some models [3] have suggested that linkage between polymorphisms subject to temporally variable selection can allow for long-term persistence of both alleles at multiple sites. Taken together, we suspect *D. melanogaster* satisfies several key features required for the long-term maintenance of balanced polymorphisms due to temporal (and spatial) variation in selection pressures. Nonetheless, how do we account for the observation that these polymorphisms have been possibly maintained across different continents with clear differences in climate and between species with different ecologies [107]?

The long-term persistence of these adaptively oscillating polymorphisms across populations, continents, and species suggests that these polymorphisms contribute to short-term and local adaptation in response to very generalized environmental conditions. This is in contrast to the hypothesis [108] that adaptation to temperate environments in *D. melanogaster* was largely in response to novel environments, exclusively associated with life in northern, temperate locales. Rather, we speculate that the selective pressures associated with seasons in temperate environments are merely manifestations of general selective pressures resulting from cyclic population booms and busts. That is, during times of plenty, such as during the summer in temperate locales, populations rapidly expand and alleles that confer increased reproductive output or faster time to sexual maturity are strongly favored. Likewise, when population size contracts due to biotic and abiotic stressors such as those experienced during winter, alleles that confer increased stress resistance are favored.

Cyclic population booms and busts are almost certainly a perennial feature of *D. melanogaster* populations, are a likely common occurrence in highly fecund species that exploit ephemeral resources, and may be an inherent property of most species in general [108]. If true, we speculate that such species may harbor alleles that promote reproductive fitness during population growth (at the cost of somatic maintenance) and increase stress tolerance (at the cost of reproductive growth) during population contraction. Such balanced polymorphisms may be particularly common for species whose population cycles are decoupled from predictable environmental cues (e.g., photoperiod) but are rather linked to stochastic changes in resource abundance. For species such as these, including many microorganisms and invertebrates, balanced polymorphisms maintained by environmental heterogeneity through time and space may be the norm rather than the exception.

## Materials and Methods

### Fly collections

We resequenced samples of *D. melanogaster* from populations sampled over several years (2003–2010) largely during periods of peak abundance along a broad latitudinal cline in North America and during multiple time points over three consecutive years (2009 to 2011) at the Linvilla Orchard in Media, PA (39.9°N, 75.4°W). From each locality and sampling period, we collected ∼50–200 *D. melanogaster* largely by aspiration from individual fruits or baiting at strawberry fields and apple and peach orchards, established isofemale lines and collected male progeny at generation 1–5 for sequencing. One male progeny per isofemale line per population was pooled together to generate template DNA for high throughput sequencing (Table S1). The only two exceptions are the second replicate sample from Maine which was derived from wild-caught males and the sample from North Carolina which was sampled from the Drosophila Genetic Reference Panel (DGRP) inbred lines. For the DGRP population, we resequenced a pooled sample consisting of one male from each of 92 DGRP strains and used allele frequency estimates from pooled samples when estimating clinality (see [41] for more information on this sample and [45] for more information on this population). Note, there is evidence that two samples (Florida replicate 2 and post-frost Pennsylvania) show low levels of contamination with the sister species *D. simulans* (i.e., ∼ one wild caught *D. simulans* was accidentally included in our pooled sample). However, we have no evidence that the low level of contamination in two samples affects our results in any way (see Text S1).

### Sample preparation, sequencing, and bioinformatics of pooled samples

DNA libraries were prepared for sequencing on the Illumina HiSeq2000 platform. To generate these libraries, we homogenized whole, male flies in 200 µL lysis buffer (100 mM Tris-Cl, 100 mM EDTA, 100 mM NaCL, 0.5% SDS) using a motorized pestle grinder. An additional 200 µL of lysis buffer was added to each sample and the homogenate was incubated at 65°C for 30 minutes. After lysis, we added 800 µL of 2 parts 5M potassium acetate, 5 parts 6M lithium chloride solution and incubated on ice for 15 minutes to precipitate proteins. The homogenate was centrifuged for at 12 K rotations per minute (RPM) for 15 minutes at room temperature, 1 mL supernatant was transferred to a new tube, and the sample was centrifuged again at 12K RPM for 15 minutes at room temperature. To precipitate DNA, we added 800 µL of isopropanol and centrifuged the sample at 12K RPM for 15 minutes. The supernatant was discarded and the DNA pellet was washed with 70% ethanol and centrifuged at 14K RPM for 10 minutes, washed with ethanol again and centrifuged once more. The ethanol was removed and the pellet was allowed to dry at room temperature. We resuspended the pellet in 100 µL TE buffer.

DNA was prepared for Illumina sequencing by shearing, end-repair and ligation. To do so, 50 µL of DNA was mixed with an additional 50 µL of TE and this DNA was sheared to ∼500 bp using a Covaris machine. DNA was eluted to 30 µL using a QIAGEN PCR-purification kit (product number 28104). We performed end repair by incubating each sample of DNA with 5 µL T4 DNA ligation buffer (New England Biolabs [NEB] product number B0202S), 4 µL of 10 mM dNTPs, 2.5 µL T4 DNA polymerase (NEB product number M0203S), 0.5 µL Klenow large fragment (NEB product number M0210S), 2.5 µL T4 PNK (NEB product number M0201S), and 5.5 µL nuclease free water for 30 minutes at 20°C. Following incubation, DNA was purified using a QIAGEN PCR-clean up kit. Next, we performed dATP addition by incubating 32 µL of DNA with 5 µL 10× NEBuffer 2 (NEB product number B7002S), 1 µL 10 mM dATP, 3 µL Klenow Exo-minus (NEB product number M0212S), and 9 µL nuclease free water at 37°C for 30 minutes. Following incubation, DNA was purified using a QIAGEN MinElute kit (product number 28004) to a final volume of 11 µL. Sequencing adapters (custom synthesized by IDT) were ligated to DNA using T/A ligation by incubating 10 µL DNA with 2 µL T4 DNA ligation buffer, 1 µL T4 ligase (NEB product number M020S), 40 µL of 40 µM pre-annealed adapter mix and 6 µL nuclease free water for 15 minutes at 20°C followed by 65°C at 10 minutes to deactivate the DNA ligase.

Finally, we performed size-selection and PCR amplification as a final step to prepare DNA sequencing libraries. Immediately following ligation, DNA was loaded into a 2%, pre-cast SizeSelect E-Gel (Life Technologies product number G661002) and run along side a 100 bp ladder. DNA at ∼500 bp was removed from the gel into a volume of ∼15 µL nuclease free water. To amplify ligated DNA, we performed two replicate PCR reactions for each sample where we used 7.5 µL template DNA, 0.25 µL of 100 µM forward and reverse primers (custom synthesized by IDT), 0.5 µL 10 mM dNTPs, 4 µL 5× High-Fidelity buffer (NEB product number B0518S), 0.5 µL Phusion High-Fidelity DNA polymerase (NEB product number M0530S), and 5 µL nuclease free water. Note, the use of two replicate PCR reactions and a high volume of template DNA was meant to prevent PCR-jackpotting. PCR was performed by 30 sections of initial denaturation at 98°C followed by 11 rounds of 10 seconds denaturation (98°C), 30 seconds annealing (65°C), 30 seconds elongation (72°C), followed by a final elongation at 72°C for 5 minutes. DNA was purified using a QIAGEN PCR-cleanup kit.

Following PCR, DNA was quantified on a Life Technologies Qubit spectrophotometer as well as with a Agilent Bioanalyzer. Libraries were diluted to the appropriate concentration and sent to the Sequencing Service Center at the Stanford Center Genomics and Personalized Medicine for sequencing on the HiSeq 2000 platform.

Raw, paired-end 100 bp sequence reads were mapped to the *D. melanogaster* reference genome version 5.39 using *bwa* version 0.5.9-r16 [109] allowing for a maximum insert size of 800 bp and no more than 10 mismatches per 100 bp. PCR duplicates (∼5% per library) were removed using *samtools* version 0.1.18 [110] and local realignment around indels was performed using GATK version 1.4–25 [111]. We mapped SNPs and short indels (i.e., those occurring within the sequence reads) using CRISP [112], excluding reads with base or mapping quality below 10. SNPs mapping to repetitive regions such as microsatellites and transposable elements, identified in the standard RepeatMasker library for *D. melanogaster* (obtained from http://genome.ucsc.edu) were excluded from analysis as were SNPs within 5 bp of polymorphic indels. SNPs with average minor allele frequency across all populations less than 15%, with minimum per-population coverage less than 10× or maximum per-population coverage greater than 400× were removed from analysis. Finally, to ensure that the examined SNPs were not artifacts of our pooled resequencing, we removed any SNP not present in the SNP tables provided by freeze 2 of the DGRP [45] (http://www.hgsc.bcm.tmc.edu/projects/dgrp/). The inclusion of reads with read and mapping qualities greater than 10 (rather than greater than 20) is justified because we are restricting our analysis to common SNPs that have been previously identified in the DGRP. Of the 1,500,000 SNPs initially identified, ∼500,000 SNPs remained after applying these filters (Table S2). SNPs were annotated using SNPeff version 2.0.5 [113]. Short intron annotations were taken from [43]. An annotated VCF file with allele frequency calls, genic annotations, and seaonsal/clinal p- and q-values is avaible on DataDryad (doi:10.5061/dryad.v883p). Raw sequence data are available from NCBI SRA (BioProject accession PRJNA256231, and see Table S1 for accession numbers of individual libraries).

### *F_ST_* estimates

To estimate average differentiation between populations or between samples collected trough time, we calculated genome-wide average (mean) *F_ST_* between pairs of populations. *F_ST_* was calculated as,
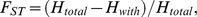
where *H_total_* is the expected heterozygosity between two populations under panmixia and *H_with_* is the heterozygosity averaged between the two populations. Estimates of heterozygosity were corrected for read depth and number of sampled chromosomes by the factor,
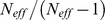
where,
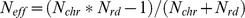
and where *N_chr_* is the number of sampled chromosomes and *N_rd_* is the number of reads at any site [114]–[116].

We performed a parametric permutation analysis to calculate the expected, genome-wide average *F_ST_* between pairs of populations under the null hypothesis of panmixia (spatial) or no allele frequency change through time (temporal) conditional on our experimental sampling design. To do so, we calculated the average allele frequency between any two pairs of populations or samples and randomly generated two estimates of allele frequency conditional on the average allele frequency, the number of reads at that site and the number of chromosomes sampled.

To calculate the proportion of SNPs where observed *F_ST_* is greater than expected by chance, we generated 500 block bootstrap samples of ∼2300 SNPs, where one SNP was drawn per 50 kb interval. The proportion of SNPs where the observed *F_ST_* distribution is greater than expected by chance is thus,
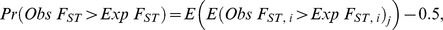
with standard deviation,
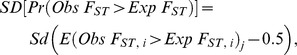
where *i* refers to the *i^th^* SNP from *j^th^* block bootstrap sample.

### Identification of seasonally and clinally varying polymorphisms

To identify clinally varying and seasonally oscillating polymorphisms, we used generalized linear models implemented in R 2.10 [117] with binomial error structure and weights proportional to the number of reads sampled at a site and the number of chromosomes sampled (see above, *N_eff_*). To identify clinal polymorphisms, we regressed allele frequency at each site (excluding all Pennsylvanian samples) on latitude (Table S1) according to the form,
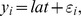
where *y_i_* is the observed allele frequencies of the *i^th^* SNP and *ε_i_* is the binomial error given the number of effective reads (see above) at the *i^th^* SNP. To identify seasonally oscillating polymorphisms, we regressed allele frequency for the three sets of spring and fall samples on a binary variable corresponding to *spring* or *fall* according to the form,
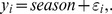
In addition, we modeled allele frequency change through time using generalized linear mixed models (GLMM) implemented in the *lme4* R package [118] and generalized estimation equations (GEE) implemented in the *geepack* R package [119]. We fit GLMMs with the model,
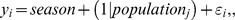
where *(1|population_i_)* corresponds to the random effect of population *j* and *ε_i_* corresponds to the binomial error. We fit GEEs with the model,
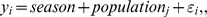
where *population_j_* corresponds to the population level strata and *ε_i_* corresponds to the binomial error fit with an autoregressive order one correlation structure. *q-q* plots (Fig. S2) demonstrate that these models (clinal and seasonal) fit the bulk of the data adequately, with the exception of the seasonal GEE model which appears to be exceedingly anti-conservative. The false discovery rate was estimated using the Benjamini & Hochberg procedure [120].

For seasonal SNPs, we estimated the cumulative selection coefficient as,
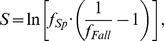
where *f_Sp_* is the average allele frequency at seasonal SNPs in the spring and *f_Fall_* is the average allele frequency at seasonal SNPs in the fall. This estimation of S is derived from a basic model of logistic growth of a beneficial allele [121], namely,
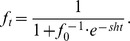
Because we do not know the specific values of heterozygosity (*h*) nor the number of generations of selections during each season (*t*), we calculate *S* as the product of *s, h*, and *t*.

### Modeling the distribution of seasonal SNPs throughout the genome

We sought to test whether seasonal SNPs were homogeneously distributed throughout the genome. To do so, we grouped the genome into bins of 1000 non-overlapping SNPs (utilizing the ∼500,000 SNPs under investigation). For each window, we calculated the number of seasonal SNPs. The number of seasonal SNPs is Poisson distributed and we examined whether the observed distribution is over-dispersed after correcting for variation in rates of recombination within chromosomes and between the autosomes and X-chromsome. To do so, we fit the generalized linear model,
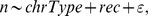
where *n* is the count of seasonal SNPs per 1000 SNPs, *chrType* is the binary classification of autosome or X-chromosome, and *rec* is the average recombination rate estimated in [64], and *ε* is the Poisson distributed error. To explicitly test if the number of seasonal SNPs is overdispersed, we used the *dispersiontest* function in the R package *AER* [122].

### Control polymorphisms and the block bootstrap

Throughout our analysis, we contrasted seasonal SNPs with control polymorphisms (Figs. 2–6). For these analyses, we identified 500 sets of control polymorphisms matched to each seasonal SNP. For each test described in the results, control polymorphisms were identified based on different sets of characteristics that have been shown, or could plausibly, influence the parameter we sought to investigate. In general, we matched seasonal SNPs to control SNPs by chromosome, recombination rate, and allele frequency in either Pennsylvania, North Carolina, North America, and/or Africa. The choice of which population to match allele frequencies was determined by the specific test. These three parameters (chromosome, recombination rate, allele frequency) correspond with many important evolutionary processes as well as genetic patterns (e.g., [123]) and therefore control SNPs will be matched to seasonal SNPs with respect to long-term evolutionary history, gene-density, and background levels of genetic variation. In general, we used as many parameters as possible while still identifying a sufficient number of control SNPs for each test and a full list of the matched characters for each test are listed in Table S3. For continuous characters, such as allele frequency, we typically rounded values so that a sufficient number of unique control sites could be identified. If no matched control SNPs were identified for a seasonal SNP, that seasonal SNP was removed from subsequent analyses.

In addition, we implemented a block-bootstrap procedure to ameliorate positive dependence of our test-statistics due to linkage disequilbrium between seasonal SNPs. We generated 500 sets of seasonal SNPs where one seasonal SNP was sampled from each 50 kb consecutive interval of the genome. This block-bootstrap yielded ∼850 SNPs that were spaced approximately every 50 Kb.

Estimates of expected values (E) of test statistics [e.g. log_2_-odds-ratios (Fig. 2C, 3B–C, 6A), *F_ST_* (Fig. 2D), probability (Fig. 4B)] and standard deviations (SD) about those expected values were calculated as,
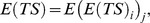


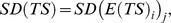
where *i* refers to control bootstrap set *i* and *j* refers to block bootstrap set *j* of any test-statistic, *TS*.

### Power calculations

To calculate statistical power of our experiment and to estimate the expected number of SNPs that are likely to vary repeatedly between seasons and along the cline we used Monte Carlo simulations based on the observed changes in allele frequency between spring and fall at seasonal SNPs or Maine and Florida at clinal SNPs. We calculated statistical power to detect seasonal SNPs as the probability of rejecting the null hypothesis of no repeatable change in allele frequency between spring and fall over three years given our sampling effort (e.g., number of chromosomes from nature and distribution of read depths in our Pennsylvanian samples) at α<∼1e-5, corresponding to observed seasonal q-value of 0.3, conditional on *S*, the cumulative change in allele frequency between seasons calculated from the logistic function. Similarly, we calculated statistical power to detect clinal SNPs as the probability of rejecting the null hypothesis of no change in allele frequency with latitude given our sampling effort at α<0.02, corresponding to the observed clinal q-value of 0.1, conditional on beta, the slope of the relationship between allele frequency and latitude. The expected number of seasonally (clinally) varying SNPs is then, the number of observed seasonal (clinal) SNPs at a particular value of S (beta) divided by the power to detect a seasonal (clinal) SNP at a selection coefficient S (beta).

### Comparison with D. simulans

To estimate the extent of trans-specific polymorphism between *D. melanogaster* and *D. simulans*, we used *D. simulans* haplotype data available from the DPGP [124] (http://www.dpgp.org/). First, we remapped raw shot-gun sequences of each *D. simulans* strain (GenBank accessions AASS00000000 - AASW00000000) to the latest release of the *D. simulans* reference genome [125] with *bwa* version 0.5.9-r16 using the *bwa-sw* method.

To convert the genomic coordinate system of the new *D. simulans* genome to the *D. melanogaster* genome, we generated a lift-over file using *lastz* [126] and components of the UCSC genome-browser toolkit [127]. Gap parameters corresponded to those used to generate the lift-over file between the first generation *D. simulans* genome and the *D. melanogaster* genome (http://hgdownload.soe.ucsc.edu/goldenPath/dm3/vsDroSim1/). The lift-over file to translate the coordinate system of the second generation *D. simulans* genome to the *D. melanogaster* version 5 genome is available on Data Dryad (doi:10.5061/dryad.v883p).

We calculated average pairwise distance between *D. melanogaster* and *D. simulans* haplotypes at seasonal SNPs that were polymorphic in both species and shared the same two alleles by state. We calculated average pairwise distance at two windows surrounding seasonal SNPs, ±1–250 bp. Note, we excluded the focal, seasonal SNP. Pairwise distance calculations were performed using the *ape* [128] package in R.

### Forward genetic simulations

To simulate genome-wide allele frequency change due to cyclic changes in population size and selection at seasonally adaptive polymorphisms, we used a modified version of the forward genetic simulation software SLiM [63]. Source code for the modified version of SLiM is available upon request. In these simulations, we modeled a 20 Mb chromosome with constant recombination rate of 2 cM/Mb. For all simulations, we seeded the chromosome with 500 neutral mutations randomly placed along the chromosome all starting at 50% initial allele frequency and in complete linkage equilibrium. The number of loci under selection varied between 0 and 30 and loci under temporally heterogeneous selection were placed equidistantly along the chromosome. Selection coefficients for each selected locus were set to produce adaptive oscillations between 40 and 60% frequency every 2 (simulated ‘winter’) and 10 (simulated ‘summer’) generations. Genotypic state was assigned randomly to each simulated diploid genome at each selected locus. Population size varied over the course of each simulation. Populations grew exponentially each ‘summer’ to a maximum population size of 10^5^ over 10 generations. Population size instantaneously crashed at the start of winter to between 5 and 10^4^ individuals and was held constant for two generations. Simulations were run for 100 generations and *F_ST_* was estimated from the last three summer-winter cycles.

### Truncation selection model

To estimate the upper bound of the number of loci that could plausibly respond to seasonally variable selection, we modeled a simple truncation selection scenario. For these models we calculated the expected number of winter adaptive alleles in the fall and the spring as the sum of average allele frequencies of the winter alleles in our fall and spring samples. If the oscillating alleles segregate independently, the variance in the number of winter alleles at any given time follows a Poisson distribution with mean and variance equal to the expected number of winter alleles. Therefore, the proportion of the population in the selected tail over winter is the probability of sampling the expected number of winter alleles in the spring from a Poisson distribution with mean equal to the number of winter alleles in the fall. To vary the number of independently oscillating polymorphisms in the spring and fall, we sub-sampled the number of oscillating polymorphisms 500 times for a range of values.

## Supporting Information

Figure S1 Genomic turnover through space and time – average *F_ST_*. Proportion of SNPs where average *F_ST_* among populations sampled along the cline (A) and through time (B) is greater than expected by chance conditional on our sampling design and panmixia among spatially separated populations or no allele frequency change through time, respectively. Lines represent the predicted values of Prop(Fst_Obs_>Fst_Exp_) for the (A) linear relationship between Prop(Fst_Obs_>Fst_Exp_) and difference latitude and (B) from non-linear relationship (y = ab^X^) between Prop(Fst_Obs_>Fst_Exp_) and difference in months. Points represent mean *F_ST_*, error bars represent 95% confidence intervals based on blocked-bootstrap resampling.
(TIF)

Figure S2 *q-q* plots and congruence of GLM, GLMM and GEE models. (A–C) Standard q-q plots of *p-values* of GLM, GLMM and GEE models, respectively. q-q plots show that GLM and GLMM models fit the bulk of the genome well whereas GEE models appear to be anti-conservative. (D) log_2_(odds-ratio) that the top 1750 seasonal SNPs identified with the GLM model are among the top 1750 seasonal SNPs identified with the GLMM model. (E) log_2_(odds-ratio) that the top 1750 seasonal SNPs identified with the GLM model are among the top 1750 seasonal SNPs identified with the GEE model.
(TIF)

Figure S3 Genomic turnover through time excluding SNPs within 1 Kb of seasonal SNPs. (A) Genome-wide average *F_ST_* between samples of flies collected through time, excluding SNPs within 1 Kb of seasonal SNPs. (B) Proportion of SNPs where *F_ST_* between pairs of samples collected through time is greater than expected by chance given the null hypothesis of no allele frequency change through time and our sampling design. Solid line represents predicted relationship between genome-wide *F_ST_* and time excluding SNPs within 1 Kb; dashed line represents predicted relationship between genome-wide *F_ST_* for all common SNPs and time. The similarity between the solid and dashed line demonstrates that SNPs near seasonal SNPs are not driving genome-wide patterns of *F_ST_* through time. Lines represent the predicted values of Fst (A) and Prop(Fst_Obs_>Fst_Exp_) (B) from non-linear regression (y = ab^X^). Points represent mean *F_ST_*, error bars represent 95% confidence intervals based on blocked-bootstrap resampling.
(TIF)

Figure S4 Enrichment among cosmopolitan inversions. Log_2_ odds ratio that seasonal SNPs are enriched among the large cosmopolitan inversions relative to control polymorphisms. Inversion breakpoints are defined as ±2.5 Mb from the proximal or distal breakpoints. Error bars represent 95% confidence intervals based on blocked bootstrap resampling.
(TIF)

Figure S5 Spatial *F_ST_* and clinal *q*-value. Scatter plot of the relationship between spatial *F_ST_* (x-axis) and –log_10_(clinal *q*-value). Colors of the hexagons represent the density of points in that interval.
(TIF)

Figure S6 Power to detect clinal SNPs. Power to detect clinal SNPs (black line) is moderate and we estimate that we have identified ∼50% (red line) of all SNPs that change in frequency monotonically with latitude (black line).
(TIF)

Figure S7 Site frequency spectrum of seasonal samples. Unfolded site frequency spectrum of spring (blue) and fall (red) samples from 2009–2010 (A) and 2010–2011 (B). Solid lines represent observed site frequency spectra, dashed lines represent simulated spring site frequency spectra conditional on one generation of bottleneck to 20 individuals and dotted lines represent simulated spring site frequency spectra conditional on two generations of bottleneck to 20 individuals. The increase in low frequency alleles in the spring 2010 sample (B, blue line) is due to the high coverage of this library. Site frequency spectra only included SNPs with allele frequencies greater than 2/(read depth) or less than 1–2/(read depth) to account for sequencing errors.
(TIF)

Table S1 Population sampling locales.
(DOCX)

Table S2 Basic SNP statistics.
(DOCX)

Table S3 Table of control characteristics.
(DOCX)

Text S1 Assessing the possibility of contamination with wild caught D. simulans. Discussion of previously identified clinal polymorphisms in relation to clinal resequencing described here.
(DOCX)
