# Supplementary figures and images for: Genomic Evidence of Rapid and Stable Adaptive Oscillations over Seasonal Time Scales in Drosophila
Source: PLoS Genet. 2014 Nov 6;10(11):e1004775. doi: 10.1371/journal.pgen.1004775 (PMC4222749; doi:10.1371/journal.pgen.1004775)

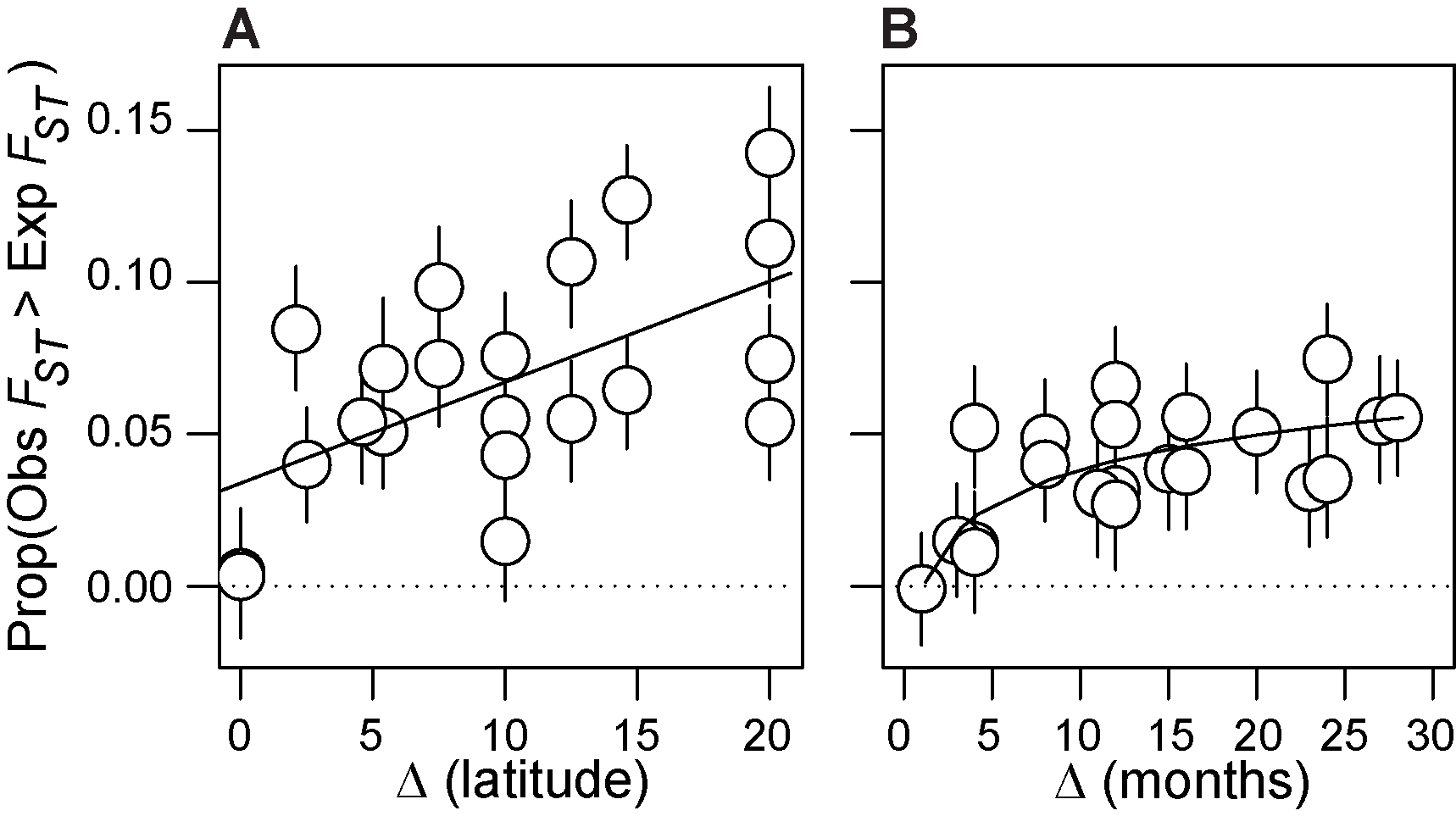

Supplement: Figure S1 — Genomic turnover through space and time – average FST. Proportion of SNPs where average FST among populations sampled along the cline (A) and through time (B) is greater than expected by chance conditional on our sampling design and panmixia among spatially separated populations or no allele frequency change through time, respectively. Lines represent the predicted values of Prop(FstObs>FstExp) for the (A) linear relationship between Prop(FstObs>FstExp) and difference latitude and (B) from non-linear relationship (y = abX) between Prop(FstObs>FstExp) and difference in months. Points represent mean FST, error bars represent 95% confidence intervals based on blocked-bootstrap resampling. (TIF) [file pgen.1004775.s001.tif]

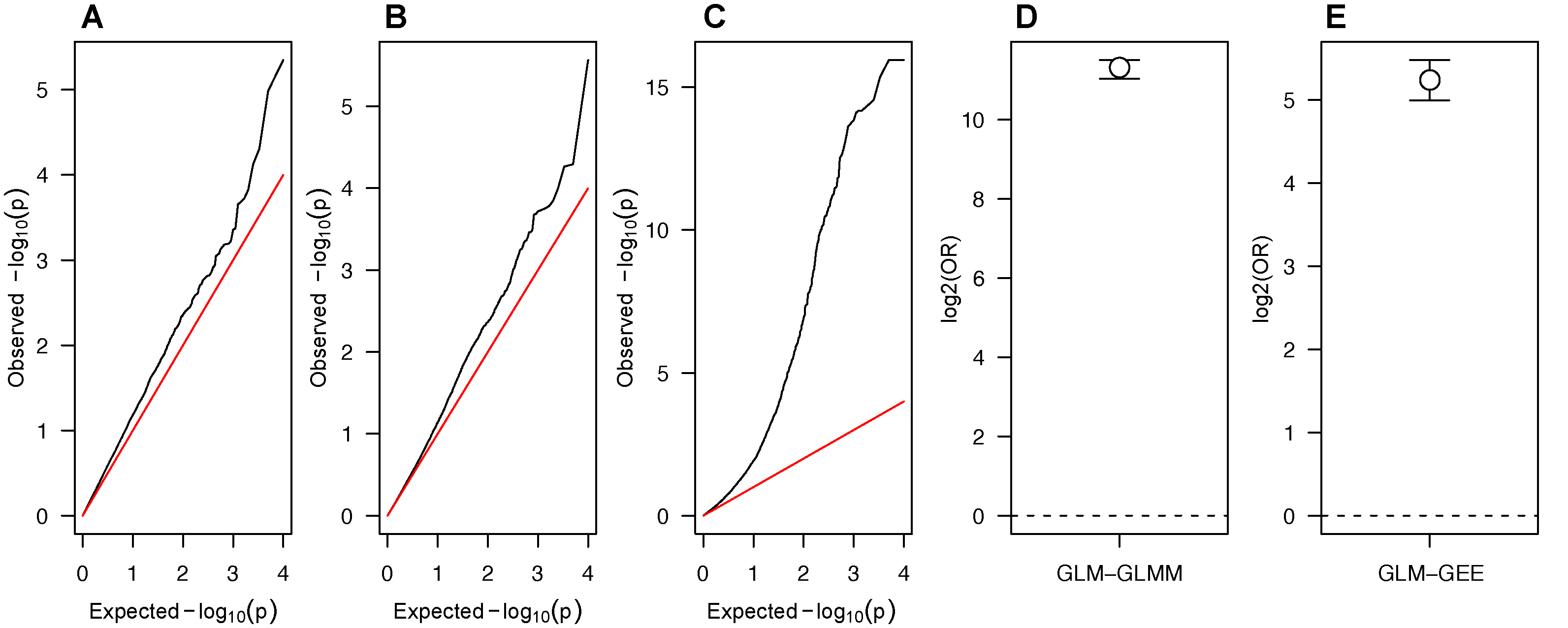

Supplement: Figure S2 — q-q plots and congruence of GLM, GLMM and GEE models. (A–C) Standard q-q plots of p-values of GLM, GLMM and GEE models, respectively. q-q plots show that GLM and GLMM models fit the bulk of the genome well whereas GEE models appear to be anti-conservative. (D) log2(odds-ratio) that the top 1750 seasonal SNPs identified with the GLM model are among the top 1750 seasonal SNPs identified with the GLMM model. (E) log2(odds-ratio) that the top 1750 seasonal SNPs identified with the GLM model are among the top 1750 seasonal SNPs identified with the GEE model. (TIF) [file pgen.1004775.s002.tif]

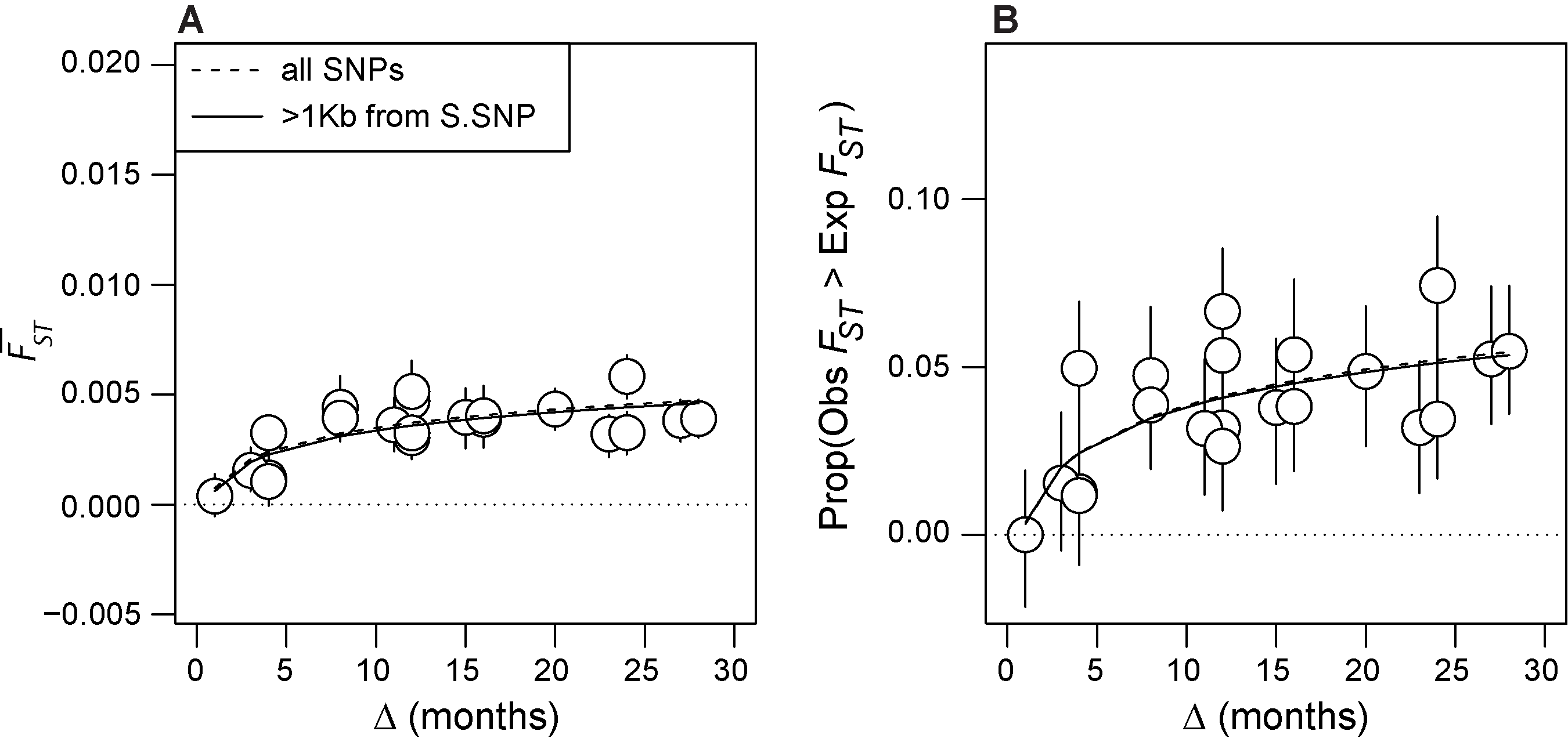

Supplement: Figure S3 — Genomic turnover through time excluding SNPs within 1 Kb of seasonal SNPs. (A) Genome-wide average FST between samples of flies collected through time, excluding SNPs within 1 Kb of seasonal SNPs. (B) Proportion of SNPs where FST between pairs of samples collected through time is greater than expected by chance given the null hypothesis of no allele frequency change through time and our sampling design. Solid line represents predicted relationship between genome-wide FST and time excluding SNPs within 1 Kb; dashed line represents predicted relationship between genome-wide FST for all common SNPs and time. The similarity between the solid and dashed line demonstrates that SNPs near seasonal SNPs are not driving genome-wide patterns of FST through time. Lines represent the predicted values of Fst (A) and Prop(FstObs>FstExp) (B) from non-linear regression (y = abX). Points represent mean FST, error bars represent 95% confidence intervals based on blocked-bootstrap resampling. (TIF) [file pgen.1004775.s003.tif]

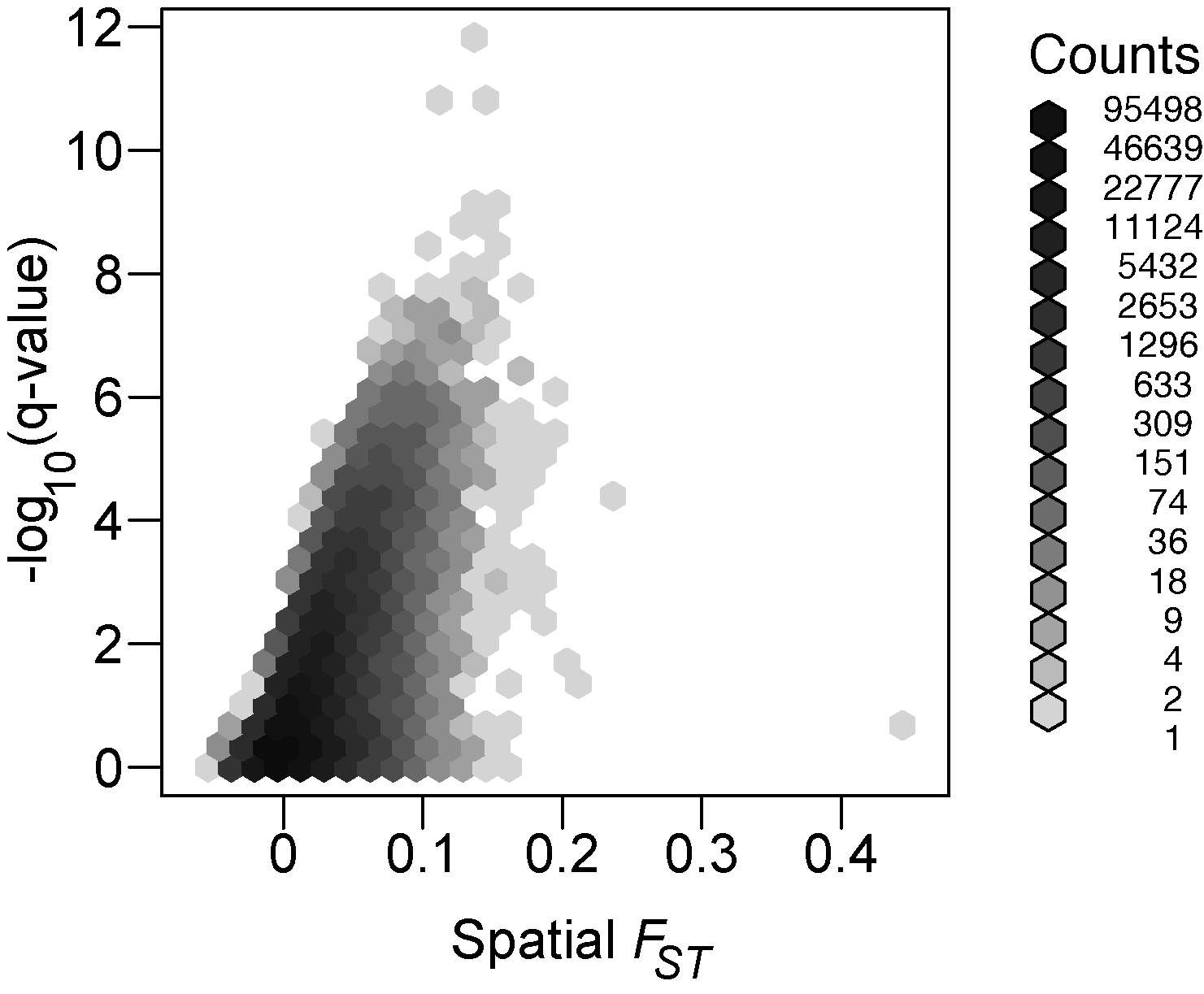

Supplement: Figure S5 — Spatial FST and clinal q-value. Scatter plot of the relationship between spatial FST (x-axis) and –log10(clinal q-value). Colors of the hexagons represent the density of points in that interval. (TIF) [file pgen.1004775.s005.tif]

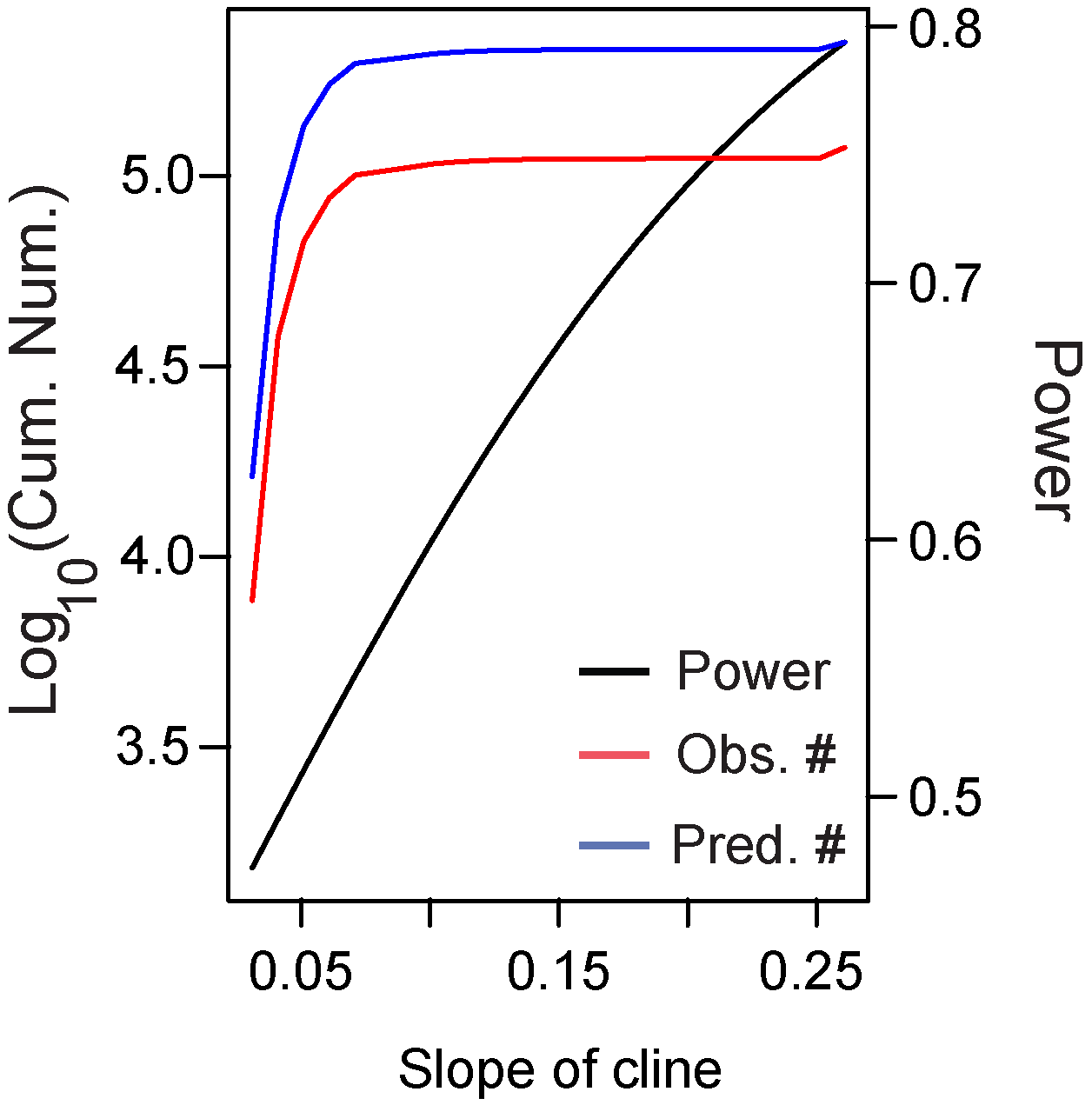

Supplement: Figure S6 — Power to detect clinal SNPs. Power to detect clinal SNPs (black line) is moderate and we estimate that we have identified ∼50% (red line) of all SNPs that change in frequency monotonically with latitude (black line). (TIF) [file pgen.1004775.s006.tif]

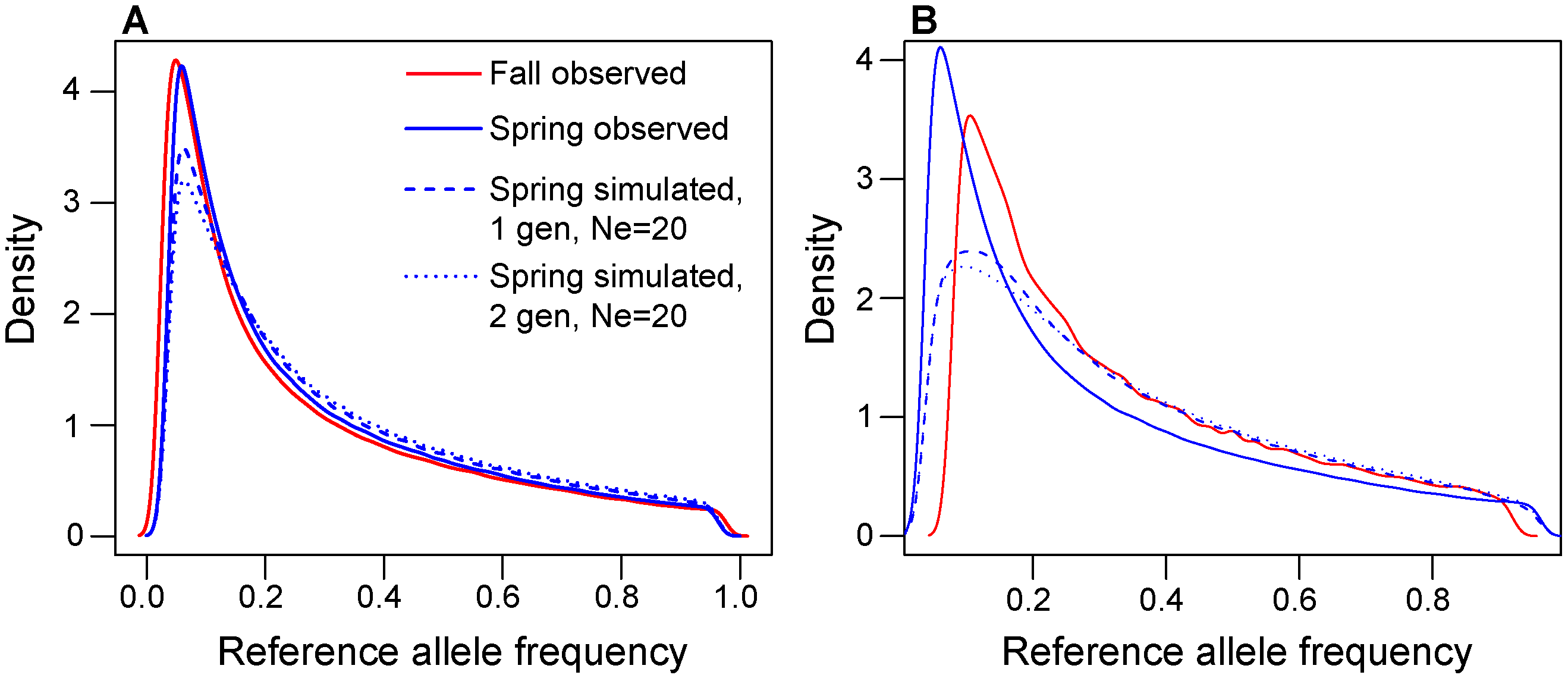

Supplement: Figure S7 — Site frequency spectrum of seasonal samples. Unfolded site frequency spectrum of spring (blue) and fall (red) samples from 2009–2010 (A) and 2010–2011 (B). Solid lines represent observed site frequency spectra, dashed lines represent simulated spring site frequency spectra conditional on one generation of bottleneck to 20 individuals and dotted lines represent simulated spring site frequency spectra conditional on two generations of bottleneck to 20 individuals. The increase in low frequency alleles in the spring 2010 sample (B, blue line) is due to the high coverage of this library. Site frequency spectra only included SNPs with allele frequencies greater than 2/(read depth) or less than 1–2/(read depth) to account for sequencing errors. (TIF) [file pgen.1004775.s007.tif]
