# Supplementary material for: Genomic Evidence of Rapid and Stable Adaptive Oscillations over Seasonal Time Scales in Drosophila
Source: PLoS Genet. 2014 Nov 6;10(11):e1004775. doi: 10.1371/journal.pgen.1004775 (PMC4222749; doi:10.1371/journal.pgen.1004775)
Supplement: Table S1 — Population sampling locales. (DOCX) [file pgen.1004775.s008.docx]

**Supplemental table 1. List of populations**

| Population | Lat. | Collection date | Chr. num (A,X)^1^ | Median read depth^4^ | SRA accession |
| --- | --- | --- | --- | --- | --- |
| Florida (rep 1) | 25.5 | 7/2008 & 7/2010 | 78, 39 | 69 | SRX661832 |
| Florida (rep 2) | 25.5 | 12/2010 | 96, 48 | 42 | SRX661833 |
| Georgia | 30.9 | 7/2008 | 102, 51 | 118 | SRX661834 |
| South Carolina | 33 | 7/2008 & 7/2010 | 96, 48 | 99 | SRX661835 |
| North Carolina^2^ | 35.5 | (5-10)/2003^3^ | 92, 92 | 43 | SRX661836 |
| Pennsylvania | 40 | 7/2009 | 110, 55 | 216 | SRX661837 |
| Pennsylvania | 40 | 11/2009 | 148,74 | 78 | SRX661838 |
| Pennsylvania | 40 | 7/2010 | 232, 116 | 29 | SRX661839 |
| Pennsylvania | 40 | 11/2010 | 66, 33 | 89 | SRX661840 |
| Pennsylvania | 40 | 7/2011 | 150, 75 | 80 | SRX661841 |
| Pennsylvania | 40 | 10/2011 | 94, 47 | 85 | SRX661842 |
| Pennsylvania | 40 | 11/2011 (post-frost) | 100,50 | 81 | SRX661843 |
| Maine (rep 1) | 45.5 | 10/2009 | 172, 86 | 105 | SRX661844 |
| Maine (rep2) | 45.5 | 10/2009 | 150, 75 | 25 | SRX661845 |

^1^ Numbers refer to the number of autosomes (A) and sex chromosomes (X) sampled from each population

^2^ See Mackay *et* *al*. [1] for the provenance of the DGRP

^3^ These flies were collected between May and October in 2003 (TFC Mackay, personal communication)

^4^ Median read depth of autosomes.

[1] Mackay TF, Richards S, Stone EA, Barbadilla A, Ayroles JF, et al. (2012) The *Drosophila melanogaster* Genetic Reference Panel. Nature 482: 173-178.
