# Supplementary material for: Genomic Evidence of Rapid and Stable Adaptive Oscillations over Seasonal Time Scales in Drosophila
Source: PLoS Genet. 2014 Nov 6;10(11):e1004775. doi: 10.1371/journal.pgen.1004775 (PMC4222749; doi:10.1371/journal.pgen.1004775)
Supplement: Table S2 — Basic SNP statistics. (DOCX) [file pgen.1004775.s009.docx]

**Supplemental table 2. SNP statistics**

|  | Number of SNPs remaining after filter |
| --- | --- |
| Total identified | 2,727,167 |
| Exclude repetitive regions | 2,566,826 |
| MAF > 0.15 | 1,119,398 |
| Greater than 5 bp from indel | 1,104,461 |
| Polymorphic in DGRP | 914,959 |
| Read depth > 10X & <400X | 557,987 |
| **Total used in analysis** | 557,987 |
