# Supplementary material for: Genomic Evidence of Rapid and Stable Adaptive Oscillations over Seasonal Time Scales in Drosophila
Source: PLoS Genet. 2014 Nov 6;10(11):e1004775. doi: 10.1371/journal.pgen.1004775 (PMC4222749; doi:10.1371/journal.pgen.1004775)
Supplement: Table S3 — Table of control characteristics. (DOCX) [file pgen.1004775.s010.docx]

**Supplemental table 3. Control polymorphism factors**.

| Analysis: | Genic element | Temporal  Fst decay | Inversion | Spatial Fst enrichment | Clinal q enrichment | Post-frost | Phenotype enrichment | Trans-specific | Average pairwise distance | Presence in Africa |
| --- | --- | --- | --- | --- | --- | --- | --- | --- | --- | --- |
| Figure | 2C | 2D | 2E, S3 | 3B | 3C | 4B | 5A-D | 6A | 6B | NA |
| Qual. filter^1^ | X | X | X | X | X | X | X | X | X | X |
| Chromosome | X | X | X | X | X | X | X | X | X | X |
| Rec. Rate^2^ | X | X | X | X | X |  | X | X | X | X |
| PA freq. | X | X | X | X | X | X |  |  |  |  |
| DGRP freq. |  |  |  |  |  |  |  |  |  |  |
| African freq. |  |  |  |  |  |  |  | X |  |  |
| Inversion |  |  |  |  |  |  |  |  |  |  |
| North Am. freq. |  |  |  | X | X |  |  |  |  |  |
| Pre – ave. delta |  |  |  |  |  | X |  |  |  |  |
| Genic |  |  |  |  |  |  |  | X |  |  |
| Synonymous |  |  |  |  |  |  |  | X |  |  |
| Informative *D. sim*. reads |  |  |  |  |  |  |  | X |  |  |
| Transspecific |  |  |  |  |  |  |  |  |  |  |
| Common in Africa |  |  |  |  |  |  |  |  |  |  |

^1^ Qual. filter includes read depth filters, distance to indels, and presence in the DGRP as described in the Materials and Methods.

^2^ Recombination rate was rounded to the nearest integer.
