# Supplementary material for: Genomic Evidence of Rapid and Stable Adaptive Oscillations over Seasonal Time Scales in Drosophila
Source: PLoS Genet. 2014 Nov 6;10(11):e1004775. doi: 10.1371/journal.pgen.1004775 (PMC4222749; doi:10.1371/journal.pgen.1004775)
Supplement: Text S1 — Assessing the possibility of contamination with wild caught D. simulans. Discussion of previously identified clinal polymorphisms in relation to clinal resequencing described here. (DOCX) [file pgen.1004775.s011.docx]

**Supplemental Information**

**Assessing the possibility of contamination with wild caught *D. simulans***. We sought to assess the possibility that the observed adaptive oscillations could be generated by a limited number of wild caught *D. simulans* being included in our samples of *D. melanogaster*. In our sampling scheme, such contamination could be particularly problematic because *D. simulans* is rare in the focal Pennsylvanian orchard in the spring but is the dominant species in the fall [1]. This raises the possibility that shifts in allele frequency at seasonal SNPs could be generated by *D. simulans* alleles consistently becoming more abundant in the fall samples.

          To assess this possibility, we first calculated the frequency of SNPs that are fixed for alternate alleles between *D. melanogaster* and *D. simulans* (kindly provided by M. Kapun). Two samples, (Florida replicate 2 and the post-frost Pennsylvania) show ~1-2% contamination; the remaining samples show no evidence of contamination (i.e., they are fixed for the *D. melanogaster* allele). Nonetheless, we sought to examined whether undetectable levels of contamination could drive oscillations at the identified seasonal SNPs. To test this, we calculated the proportion of seasonal SNPs where the fall allele is identical by state to the *D. simulans* allele. We contrasted this proportion to 500 sets of control SNPs matched to the seasonal SNPs by chromosome, recombination rate, and average allele frequency. The log2(odds ratio) that the fall allele is identical by state to the *D. simulans* allele is 0.08 and is not significantly different from zero (p = 0.144). Therefore, any possible contamination by *D. simulans* is exceedingly unlikely to drive patterns of allele frequency change at the observed seasonal SNPs.

**Previously identified clinal SNPs.** We tested if previously identified North American clinal variants show signals of clinality in our resequencing data. To do so, we performed a literature search and identified all reported genes with clinal variants (either allozymes or SNPs) in North America. Of the 10 genes that have been identified previously as clinal, 9 show signals of clinality in our data as indicated by a clinal polymorphism within 1kb of the gene (clinal in our data: *sgg* [2]*, InR* [3], *UGPase* [4]*, Pgm* [4,5]*, Gdph* [4]*, Treh* [4], *Adh* [6]*, cpo* [7]*, mth* [8]*,;* not clinal in our data: *Gdph* [4]).

1 Schmidt, P.S. Evolution and mechanisms of insect reproductive diapause: A plastic and pleiotropic life history syndrome. In *Mechanisms of Life History Evolution*, T. Flatt and A. Heyland, Eds. Oxford University Press, Oxford, (2011).

2 Rand, D. M., Weinreich, D. M., Lerman, D., Folk, D. & Gilchrist, G. W. Three Selections Are Better Than One: Clinal Variation of Thermal QTL from Independent Selection Experiments in Drosophila. *Evolution* **64**, 2921-2934, (2010)

3 Paaby, A. B., Blacket, M. J., Hoffmann, A. A. & Schmidt, P. S. Identification of a candidate adaptive polymorphism for Drosophila life history by parallel independent clines on two continents. *Molecular Ecology* **19**, 760-774 (2010)

4 Sezgin, E. *et al.* Single-locus latitudinal clines and their relationship to temperate adaptation in metabolic genes and derived alleles in *Drosophila melanogaster*. *Genetics* **168**, 923-931 (2004)

5 Duvernell, D. D. & Eanes, W. F. Contrasting molecular population genetics of four hexokinases in *Drosophila melanogaster*, *D. simulans* and *D. yakuba*. *Genetics* **156**, 1191-1201 (2000)

6 Berry, A. & Kreitman, M. Molecular analysis of an allozyme cline: alcohol dehydrogenase in *Drosophila melanogaster* on the east coast of North America. *Genetics* **134**, 869-893 (1993)

7 Schmidt, P. S. *et al.* An amino acid polymorphism in the *couch potato* gene forms the basis for climatic adaptation in *Drosophila melanogaster*. *Proc Natl Acad Sci U S A* **105**, 16207-16211, (2008)

8 Duvernell, D. D., Schmidt, P. S. & Eanes, W. F. Clines and adaptive evolution in the *methuselah* gene region in *Drosophila melanogaster*. *Mol Ecol* **12**, 1277-1285, (2003)
